# Metabolomics in hepatocellular carcinoma: From biomarker discovery to precision medicine

**DOI:** 10.3389/fmedt.2022.1065506

**Published:** 2023-01-04

**Authors:** Xingyun Wu, Zihao Wang, Li Luo, Dan Shu, Kui Wang

**Affiliations:** ^1^ West China School of Basic Medical Science & Forensic Medicine, Sichuan University, Chengdu, China; ^2^ Department of Gastrointestinal Surgery, West China Hospital, Sichuan University, Chengdu, China; ^3^ Center for Reproductive Medicine, Department of Gynecology and Obstetrics, West China Second University Hospital, Sichuan University, Chengdu, China; ^4^ Key Laboratory of Birth Defects and Related Diseases of Women and Children (Sichuan University), Ministry of Education, Chengdu, China; ^5^ School of Bioscience and Technology, Chengdu Medical College, Chengdu, China

**Keywords:** hepatocellular carcinoma, metabolomics, precision medicine, biomarkers, multi-omics

## Abstract

Hepatocellular carcinoma (HCC) remains a global health burden, and is mostly diagnosed at late and advanced stages. Currently, limited and insensitive diagnostic modalities continue to be the bottleneck of effective and tailored therapy for HCC patients. Moreover, the complex reprogramming of metabolic patterns during HCC initiation and progression has been obstructing the precision medicine in clinical practice. As a noninvasive and global screening approach, metabolomics serves as a powerful tool to dynamically monitor metabolic patterns and identify promising metabolite biomarkers, therefore holds a great potential for the development of tailored therapy for HCC patients. In this review, we summarize the recent advances in HCC metabolomics studies, including metabolic alterations associated with HCC progression, as well as novel metabolite biomarkers for HCC diagnosis, monitor, and prognostic evaluation. Moreover, we highlight the application of multi-omics strategies containing metabolomics in biomarker discovery for HCC. Notably, we also discuss the opportunities and challenges of metabolomics in nowadays HCC precision medicine. As technologies improving and metabolite biomarkers discovering, metabolomics has made a major step toward more timely and effective precision medicine for HCC patients.

## Introduction

Hepatocellular carcinoma (HCC) remains a global health burden, accounting for approximately 1 million newly emerged cases annually (1, 2). With an ever-increasing incidence and poor prognosis, HCC ranks as the second leading cause of cancer deaths worldwide (3). Driven by various genetic and environmental factors, the majority of HCC develops and progresses with the background of chronic hepatitis and liver cirrhosis (4). Most risk-factors for HCC have been identified nowadays, including chronic infection with hepatitis B virus (HBV) or hepatitis C virus (HCV), liver cirrhosis, non-alcoholic fatty liver disease (NAFLD), diabetes, and exposure to toxins such as aflatoxins and aristolochic acid (5–8). Although the understanding of HCC etiology has largely improved recently, the details of molecular alterations with HCC progression remain ill-defined, especially in a metabolic perspective. Accumulating evidence suggests that metabolites play essential roles in the malignant development of HCC (9, 10). The dynamic metabolic phenotype can reveal what is currently occurring in HCC patients (11). Therefore, there is an urgent need for comprehensive understanding of metabolic characteristics for HCC progression.

The early diagnosis determines a better overall survival of HCC patients (12). Because of the asymptomatic nature of early-stage HCC, most HCC patients are firstly diagnosed at advanced stage (13). The current screening of possible HCC patients mainly relies on liver ultrasonography and serum α-fetoprotein (AFP) testing (14). However, due to the limited sensitivity and accuracy of ultrasonography, small HCC lesions are difficult to be distinguished from cirrhosis nodules (15). Even though ultrasonography can be combined with AFP testing, unavoidable false positives still remain a major trouble in HCC diagnosis (16, 17). Limited and less-specific biomarkers obstruct timely diagnosis and treatment for HCC patients. Nowadays, emerging studies have being focusing on identifying novel biomarkers for HCC.

Metabolomics is a powerful technology for profiling metabolic features by qualitative and quantitative analysis of various metabolites in given samples (18). The contents of identified metabolites are influenced by sample types and analytical methods. Common biological materials for clinical metabolomics studies include tissues, biofluids (including blood and urine), and feces (9, 19, 20). Nuclear magnetic resonance (NMR) and mass spectrometry (MS) are two widely applied analytic platforms for metabolomics analysis, with the advantage of high throughput, high resolution, and low invasion (21). Biologically, in-depth analysis of metabolic profiles in clinical samples enables better understanding of the metabolic features, disease progression and other clinically related aspects (22). Recently, improvements in analytical instruments and strategies promote the biomedical and clinical applications of metabolomics approaches, especially in the field of cancer therapy. Increasing studies employ metabolomics as a powerful tool to uncover metabolic features, metabolite biomarkers, and driving factors during HCC tumorigenesis. In particular, metabolomics holds great promise in guiding precision medicine for HCC patients to maximize therapeutic efficacy and minimize decision failure (11, 23, 24). It is worth noting that metabolic disturbances are associated altered molecular basis of diseases, including metabolic gene/protein expressions and epigenetic regulations (25). Therefore, the integrated analysis of multi-omics data can provide comprehensive information from different molecular insights. In this regard, multi-omics analysis in an integrated framework has been proposed with great potential to facilitate biomarker discovery and treatment guidance in future clinical therapy (6, 26, 27).

Over the past decades, increasingly improved analytical strategies and devices are the foundation for emerging HCC metabolomics studies. Novel metabolites biomarkers, identified from HCC metabolomics studies can promote the improvement of HCC clinical treatment. In this review, we firstly introduce the current metabolomics technologies for metabolic profiling. Subsequently, we summarize the recent advances in metabolomics studies for HCC biomarker discovery. Notably, we highlight the application of multi-omics-based strategies containing metabolomics in biomarker discovery for HCC. These biomarkers are expected to make a major step toward more timely and effective precision medicine for HCC patients.

## Metabolomics approaches for metabolite profiling

**Figure 1:**
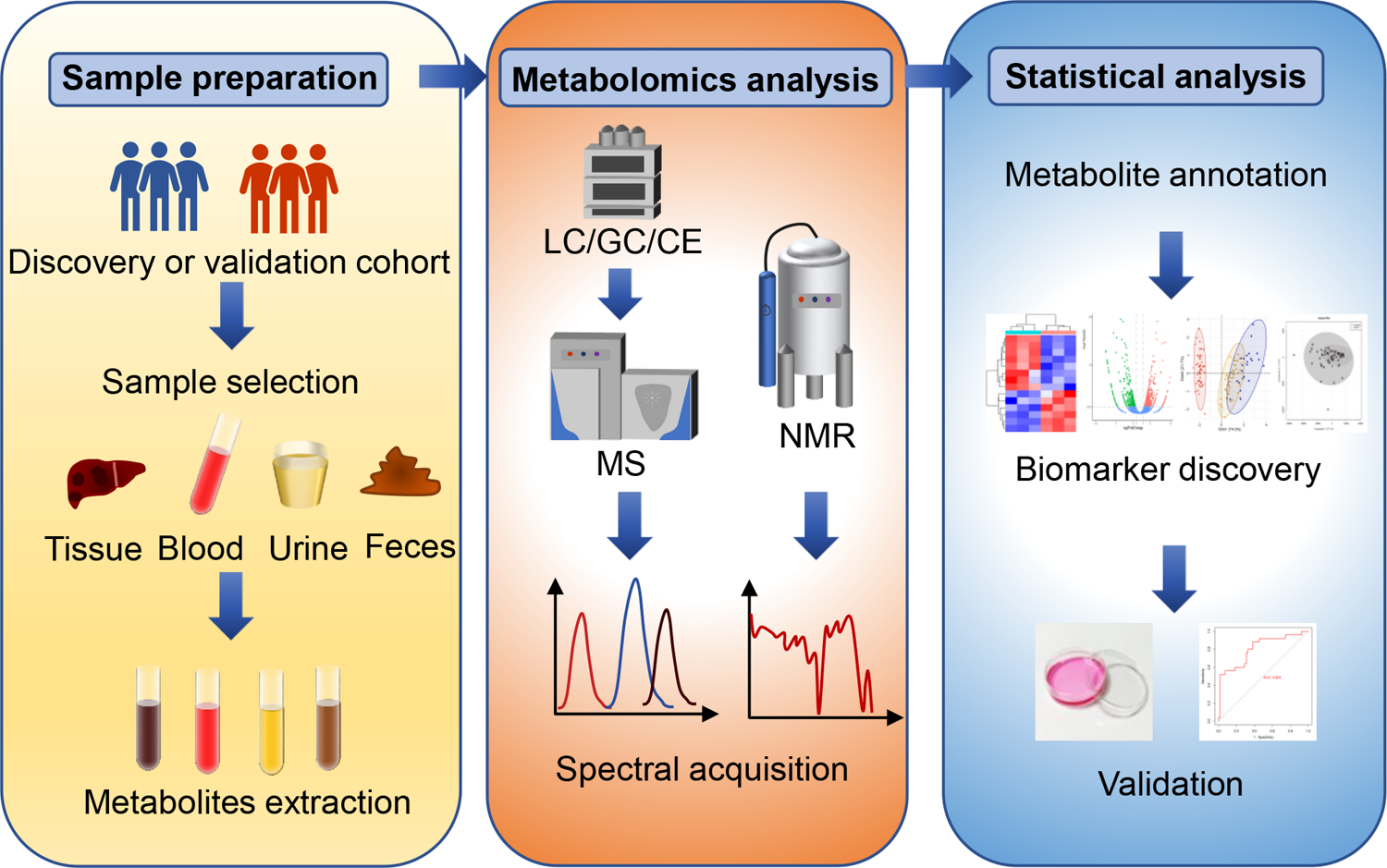
The general workflow of metabolomics analysis. The general metabolomics analysis consists of sample collection and preparation, metabolomics detection and statistical analysis. Common samples include pathological tissues, blood-derived samples, urine, and feces. Nuclear magnetic resonance (NMR) and mass spectrometry (MS) are two widely applied analytic platforms. MS is always coupled with gas chromatography (GC), liquid chromatography (LC) or capillary electrophoresis (CE) for different separation demands. The metabolic data obtained from these analytic platforms require pre-processing, metabolite identification and quantification, and statistical analysis. Common statistical analysis includes principal component analysis (PCA), partial least squares regression discriminant analysis (PLA-DA) and orthogonal partial last squares regression discriminant analysis (OPLS). Finally, receiver operating characteristic curve (ROC) analysis and *in vivo* validation can be utilized to evaluate the specificity and sensitivity of novel biomarkers.

The general workflow of metabolomics analysis consists of sample collection and preparation, analytical platforms for metabolomics, and statistical analysis of metabolomics data (Figure 1).

### Sample preparation

During sample preparation, the first issue of concern is which type of clinical materials is needed for metabolomics studies. Common samples include pathological tissues, blood-derived samples, urine, and feces (23, 28). Tissues directly exhibit metabolic alterations during disease development and progression, and represent the most persuasive material for studying mechanism of diseases (29). However, the acquisition of pathological and healthy tissues is invasive and difficult. In addition, quenching is an important step after tissue sampling, which aims to rapidly inhibit metabolism, but is dispensable for biofluids (30). Besides, fully extracting metabolites from tissues, either by solvent extraction or acid extraction, is another challenge (31). These limitations restrict the use of tissues in metabolomics. Instead, blood, urine, and feces are easy to access and low-invasion in clinical practice. Blood is described as one of the most informative materials, which carries many metabolites from local lesions or distant metastasis sites into peripheral circulation. Therefore, serum and plasma are ideal materials to identify biomarkers for early diagnosis and prognosis (32, 33). To identified more convincing metabolite biomarkers, some studies firstly identify altered metabolites in tissue samples and subsequently validate them in blood by targeted analysis. In addition, paired analysis of entering and draining blood of pathological tissues can provide *bona fide* information of tissue metabolic activity (34). This arteriovenous-based paired approach has great power to explore the pathological mechanisms which was often ignored before. For example, portal venous blood, the unique blood supply of liver originated from intestinal system, has recently been subjected to in-depth metabolomics analysis. Metabolite differences from portal venous to central venous in blood were combined with metabolite profiles in tissues and feces. This study is the first report to illustrate the metabolome characteristics in portal vein of HCC patients and highlights portal venous blood as reliable clinical materials (9). Due to the stability of biofluid-derived samples, quenching is less important considerations. Instead, immediately −80°C freezing and storage are essential for maintaining sample integrity to achieve accurate analytical results. Besides, the use of urine and feces samples in metabolomics are increasing nowadays. They include both endogenous and exogenous metabolites, which can indicate the influence of genetic and environmental factors on metabolic alterations during disease progression (35). Specifically, the fecal metabolomics studies can capture the potential interactions between the gut microbiome and disease development (36). Metabolites from gut microflora may contribute to the initiation and progression of HCC, which are promising biomarkers for diagnosis and prognostic evaluation.

It should be noted that some animal models, such as diethyl-nitrosamine (DEN)-induced HCC in mice or rats, can simulate human HCC in the typical pathological type (37). Animal models play important roles in analyzing the dynamic alterations of metabolites during hepatocarcinogenesis in pre-clinic studies. ^1^H-NMR- and MS-based metabolomics studies have been performed for the identification of metabolite biomarkers for HCC by using DEN-induced HCC model (38–40). However, other HCC animal models seem to be ignored for metabolomics studies, such as HCC transgenic mouse models (for example, c-myc and transforming growth factor *α* expression systems) and tumor xenografts (41–44). Expanding the application of metabolomics analysis in different HCC animal models is an important ongoing area of investigation.

### Analytical platforms for metabolomics studies

NMR and MS platforms are the most widely used analytical tools for metabolomics studies (21). NMR identifies metabolites based on the specific radiation generated by ^1^H or ^13^C atoms in a magnetic field, which is highly reproducible and nondestructive (45–47). ^1^H-NMR is widely applied due to its simple sample preparation and robust structure characterization ability (48). NMR has an advantage in analyzing organic molecules, especially fatty acids (49). Moreover, NMR can provide non-destructive analysis of metabolites in intact cells and organisms, which has great potential for clinical application (50). For example, proton magnetic resonance spectroscopy can dynamically track D-2-hydroxyglutarate in gliomas to monitor disease progression and predicting treatment outcomes (51). However, the low sensitivity and resolution of NMR limit its implication (52–54). In this regard, two-dimensional NMR spectroscopy has been recently developed with high resolution, which is promising to extend the application of NMR in metabolomics studies (52).

Currently, MS is the most sensitive tool for metabolomics analysis with broad coverage and high scalability. Because of the complex diversity and heterogeneity of metabolite properties, no single method can efficiently cover all metabolites at the same time (30). Therefore, MS platform is always equipped with different chromatographic columns to increase its sensitivity and resolution for specific metabolites of interest. Gas chromatography (GC), liquid chromatography (LC) and capillary electrophoresis (CE) are common separation devices to reduce sample matrix and separate individual metabolites (33, 39, 55, 56). GC-MS has a broad detect spectrum, and can well identify nonpolar and volatile metabolites, such as organic acids, sugars, and free fatty acids (FFAs) (57, 58). However, the high-energy and low vapor pressure of GC limit its application to heat-labile metabolites. Therefore, GC-MS needs to be coupled with chemical derivatization process to increase candidate stability (58). LC-MS, the most widely used tool for metabolomics analysis, is quite versatile with several different retention patterns, including reversed phase, normal phase, and hydrophilic interaction chromatography (59). LC-MS is sensitive and accurate that can be employed for metabolites with low concentrations (60). However, LC-MS works poorly with non-polar molecules. Recently, a dual derivatization LC-MS-based metabolomics has been launched as an easy-to-use strategy for quantifying nonpolar metabolites, such as FFAs. In this study, a pair of light and heavy derivatization reagents are used to label FFAs to improve their ionization efficiency, rendering these non-polar metabolites suitable for LC-MS analysis (61). CE-MS is a mature separation technique for polar metabolites. However, due to its low sensitivity and high variability, the application of CE-MS is largely limited in metabolomics studies (62).

For MS-based metabolomics analysis, the separated metabolites need to be ionized for signal collection. It is worth noting that different ionization methods will generate different metabolomic profiles (63). Electrospray ionization (ESI), electron impact ionization, and chemical ionization are common ionization methods. ESI model can softly ionize a wide spectrum of metabolites, representing the most effective ionization choice for LC-MS (64). However, ESI lacks quantitative capability, due to the ion suppression/enhancement from matrix interference. Electron impact ionization and chemical ionization methods have been developed to circumvent the interference of matrix (65). Benefiting from the rapid development of separation and ionization strategies, MS platform has steadily increased its application for metabolomics studies.

MS-based metabolomics strategies can be either targeted or nontargeted. To achieve different analytic goals, various mass analyzers are available, including Fourier transform ion cyclotron resonance (FT-ICR), orbitrap, time of flight (TOF), ion trap, and quadrupole (Q) (59, 66). Of note, these analyzers can be combined as hybrid MS systems for better performance (67–69). Nontargeted metabolomics usually utilizes FT-ICR-, TOF-, Orbitrap- or Q-TOF-based MS platforms to cover as many metabolites as possible in a single analysis (70). Early metabolomics studies often identify altered metabolites using nontargeted strategies, but lack validation. Targeted metabolomics acts as hypothesis-driven analysis and aims to qualitative and quantitative pre-defined metabolites. To achieve this attempt, tandem MS is always equipped with triple quadrupole or Q-linear ion trap to specifically identify certain molecules (69, 71). Selected reaction monitoring (SRM) and multiple reaction monitoring (MRM) are the basic signal collection devices in targeted metabolomics (72). In general, altered metabolites are screened by untargeted metabolomics, and subsequently verified by targeted analysis. It should be noted that corresponding metabolite standard is required for targeted analysis of candidate metabolites. Interestingly, a pseudo-targeted method has recently been developed for biomarker discovery based on both nontargeted and targeted platforms. Briefly, ion pairs of metabolites were figured out by nontargeted analysis. Subsequently, targeted devices were used to quantify as many metabolites as possible based on untargeted profiling information. Compared to nontargeted metabolomics, the pseudo-targeted metabolomics method can achieve better repeatability and wider linear range (73).

### Statistical analysis of metabolomics data

The metabolomics data are quite massive and variable. Therefore, sophisticated statistical analysis is required for identifying significantly altered metabolites (74). After acquiring the metabolomics data, analysis of the data distribution makes primary. Then, for hypothesis-driven analysis, Student's *t*-test and Mann–Whitney *U* test can be utilized to compare the differences between two groups. *χ*^2^ test and Fisher's exact test are suitable for categorical parameters (9). As for non-hypothesis analysis, multivariate analytic approaches are required, such as principal component analysis (PCA), partial least squares regression discriminant analysis (PLS-DA), and orthogonal partial last squares (OPLS). PCA is a common unsupervised tool for data simplification, which can capture a panel of most contributing factors by calculating dimensionless components (75). Subsequently, supervised methodologies, including PLS-DA and OPLS analysis, are carried out to produce intuitive data interpretation by the reduction of dimensionality and noise of data (76). Supervised methodologies are usually combined with unsupervised PCA to identify the predictive metabolic alterations from uncorrelated information in practice (9). Finally, the specificity and sensitivity of selected metabolites can be evaluated by receiver operating characteristic (ROC) curve analysis and *in vivo* validation. However, due to the similarity existed between the metabolic features between HCC and liver cirrhosis, a pattern recognition approach has been developed and employed for HCC metabolomics study based on sequential feature selection in combination with linear discriminant analysis (77). When combined with AFP diagnostic model, this recognition analysis is effective enough to distinguish HCC from healthy controls or liver cirrhosis than conventional data processing methods (78). Additionally, a LC-MS-based metabolomics study have established a network-based feature selection method (NFSM) to define metabolites with the most discriminant capacity of outcome prediction. In NFSM, paired biomarkers were selected to infer metabolite networks and calculate feature ratios, which were considered as presumptive pathway reactions. By using statistical significance of feature ratios, NFSM was conducted to define the key metabolites associated with different outcomes. Finally, a risk score, constituting of serum levels of paired metabolites, was established and validated to predict the overall survival of HCC post-surgery patients. Based on this approach, phenylalanine and choline were identified as a biomarker panel for prognostic evaluation of HCC patients after surgical intervention (63). With the development of analytic methodologies, metabolomics-based biomarker discovery has been speeded up for clinical practice.

## HCC metabolomics for biomarkers discovery

**Figure 2:**
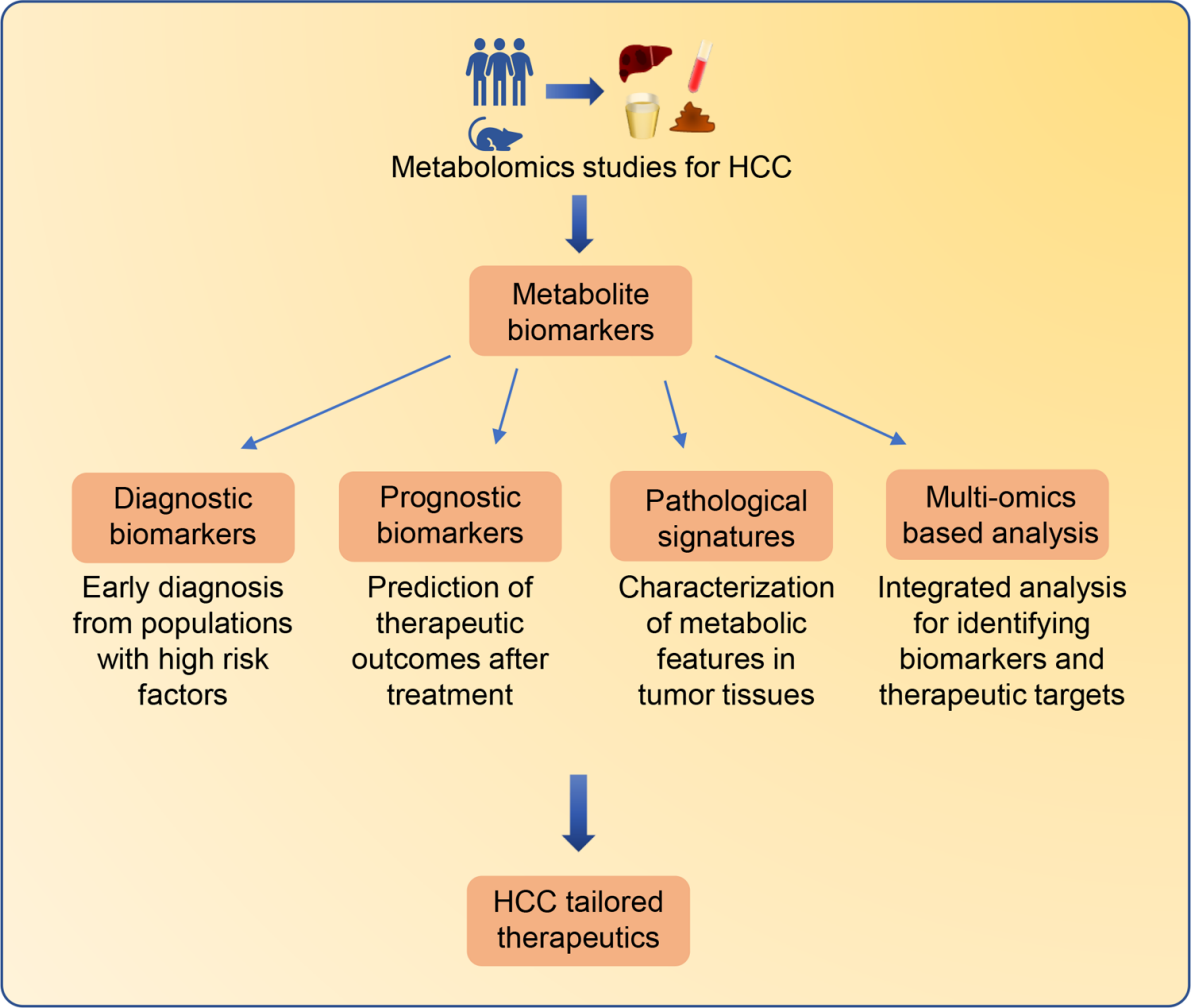
Metabolomics-based biomarkers discovery for tailored therapeutics for HCC patients. Significant altered metabolites identified from HCC metabolomics studies can be employed as biomarkers for different clinical applications. Metabolite biomarkers in biofluids can be employed for early diagnosis and prediction of therapeutic outcomes. Characterizing metabolic features in HCC tissues can improve the understanding of HCC pathological signatures. In addition, the integrated multi-omics strategies containing metabolomics can provide comprehensive information from different molecular insights for the identification of HCC biomarkers and therapeutic targets.

To date, emerging metabolomics studies have been successfully conducted to characterize the metabolic features of HCC (Supplementary Table S1). Several metabolites involved in multiple metabolic pathways have been identified as potential HCC biomarkers. In some cases, alerted metabolites in biofluids can be utilized as biomarkers for early diagnosis or prediction of the therapeutic outcomes in clinical practice. Moreover, metabolomics analysis of tumor tissues enables the discovery of novel metabolites which contribute to the development and progression of HCC. In particular, multi-omics-based strategies have been used to identify metabolic biomarkers for HCC (Figure 2).

### Metabolite biomarkers in biofluids for HCC early diagnosis

Early detection of HCC is vital for longer survival time and more available therapeutic options (79). Blood and urine, which are easy to access and simple to operation in clinical practice, serve as ideal material sources for biomarker discovery (80). A CE-MS-based metabolomics study employed 7 serum samples from DEN-induced rats to discover polar metabolites for early diagnosis of HCC. Among the 76 significant differential metabolites, 5 metabolites, including betaine, creatine, kynurenine, pipecolic acid, and one unidentified metabolite were cross-selected based on the preliminary screening of multivariate and univariate analysis. Among them, the increased ratio of creatine/betaine achieved effective classification of pre-HCC and HCC stages. This ratio also performed well in an external validation study for cirrhosis and HCC patients (39). Besides, a ^1^H-NMR-based metabolomics study was carried out to identify potential biomarkers by using urine samples from DEN-induced HCC rats. Time-dependent changes of metabolites in urine were analyzed when rats were at the 8th, 10th, 12th weeks of age. After multivariate statistical analysis, a total of 26 altered metabolites were identified in HCC model. Then, pathway enrichment analysis was performed for metabolite profiles at the 8th, 10th, and 12th weeks of age. The taurine and hypotaurine metabolism was exhibited the highest alteration. The result showed that urinary levels of creatinine, putrescine, choline, and taurine were increased, whereas the level of hippurate was decreased in HCC tissues compared with normal tissues (38). These altered metabolites are associated with HCC occurrence and suitable for HCC early diagnosis. However, these metabolites need to be further validated in HCC patients. In addition, Tan et al*.* conducted LC-MS-based metabolomics to discover potential biomarkers in serum for small HCC diagnosis. Serum from 56 DEN-induced HCC rats were collected as discovery group. Using LC-MS-based screening, 52 metabolites exhibited significant differences. Among them, several metabolites, such as taurocholic acid, lysophosphoethanolamine, and lysophosphatidylcholine, exhibited increased trends of alterations with hepatocarcinogenesis. To examine the diagnosis potential, these selected metabolites were validated in human serum from 262 patients with HCC, 76 patients with cirrhosis and 74 patients with HBV infection. The serum levels of taurocholic acid, lysophosphoethanolamine, and lysophosphatidylcholine were significantly up-regulated in HCC patients (40). Therefore, these metabolites are regarded as promising biomarkers for HCC diagnosis. These studies suggest that animal models can be employed as discovery group to identify potentially altered metabolites involved in HCC development and progression.

Biologically, in-depth analysis of serum metabolic profiles of HCC patients and healthy populations enables biomarker discovery for HCC. The level of endogenous metabolites in blood are related to the risk of HCC. An ultra-performance liquid chromatographic (UPLC)-MS-based metabolomics study used 209 serum samples to characterize metabolic features of HCC patients. Among 1960 detected metabolites, 9 metabolites showed higher peak intensities, and 28 metabolites showed significantly lower peak intensities in HCC groups. Correlations analysis revealed that the occurrence of HCC is highly associated with the increased serum levels of leucine, phenylalanine, tyrosine, arachidonic acid, and 5-hydroxyhexanoic acid (81). Besides, Wu et al*.* detected the differential metabolites in urine through chemical derivatization followed by GC-MS analysis. Among the 103 observed metabolites, 16 endogenous metabolites were significantly up-regulated in HCC patients compared with healthy controls, whereas 2 metabolites were down-regulated. Combining with AFP examination, these metabolites performed well for early diagnosis through cross-validated ROC analysis (56). This work may inspire future studies to investigate more exogenous metabolites originated from drugs or human floras as biomarkers for HCC. Non-targeted serum metabolomics profiling is regarded as a noninvasive tool to provide early diagnostic differentiation. Recently, an untargeted metabolomics study aimed to examine the association of plasma metabolites with the risk of HCC by using two prospective cohorts (108 and 55 HCC and healthy individuals, respectively) in China. UPLC-MS-based platforms have discovered 44 dysregulated metabolites between HCC patients and controls. After least absolute shrinkage and selection operator (LASSO) and ROC curve analysis, 18 metabolites (including quinolinate, glycocholic acid, and citraconate) were selected with the potential to predict HCC risk in both training and validation sets (82). To distinguish early and advanced HCC patients, an NMR-based studies used serum samples from 64 HCC patients in early (28 populations) and advanced (36 populations) disease stages for biomarker discovery. OPLS-based analysis showed the levels of alanine, glutamine, 1-methylhistidine, valine, and lysine were increased in early HCC, while N-acetylglycoproteins and glycine were decreased. Moreover, Kaplan-Meier analysis highlighted the serum tyrosine as a predictor for overall survival of early HCC patients (83). Together, this study identified a set of metabolites as potential biomarkers for early HCC. In addition to endogenous metabolites, exogenous molecules could also serve as promising biomarkers for HCC. For instance, a ^1^H-NMR-based metabolomics employed serum from 144 HCC patients and 222 healthy individuals as objects of study. A total of 285 differential metabolites were identified. Among them, serum levels of 8 metabolites were higher in HCC patients, and 15 metabolites were lower. After ROC analysis, 16 metabolites, including tyrosine, phenylalanine, citrate, glucose, propylene glycol, glutamate, valine, acerate, leucine, isoleucine, choline, N-acetylglycoproteins, unsaturated lipids, and very-low-density lipoproteins were found to be significantly associated with HCC risk. Specifically, propylene glycol, an exogenous metabolite, was a strong risk positively associated with HCC (84).

It is known that some cases of HCC arise with a background of HBV or HCV infections (85, 86). Therefore, several metabolomics studies focus on investigating the difference of blood metabolites in HCC and hepatitis patients. To distinguish HCC and HBV patients, a GC-MS-based metabolomics study investigated serum metabolites from 39 HCC patients, 49 HBV-infected patients, and 61 healthy people. Peak signals of 300 metabolites were obtained. Among them, 11 metabolites were significantly up-regulated in HCC serum compared to healthy populations and patients with HBV and liver cirrhosis. These altered metabolites in serum were proved to be associated with the stepwise of hepatocarcinogenesis from hepatitis B to HCC (87). Besides, a LC-MS-based metabolomics screening was carried out to distinguish HCC from chronic hepatitis B and liver cirrhosis. Metabolite profiling revealed that 858 and 1,720 metabolites were up-regulated and down-regulated respectively in HCC serum samples compared with HBV patients. By comparing metabolite profiling from liver cirrhosis patients, 51 differential metabolites were selected, which were altered with the progression from chronic hepatitis B to liver cirrhosis to HCC. Specifically, the levels of taurodeoxy cholic acid and 1,2-diacyl-3-β-D-galactosyl-sn-glycerol were gradually increased with liver disease progression, whereas the levels of 5-hydroxy-6E,8Z,11Z,14Z,17Z-eicosapentaenoic acid and glycyrrhizic acid were decreased (88). As for HCV-based HCC, 40 HCC patients with underlying HCV infections and 22 HCV patients were recruited for a ^1^H-NMR-based study. Individual peaks from 19 known metabolites were collected and analyzed. It is shown that the serum levels of choline and valine were increased, whereas the level of creatinine was decreased in HCC patients. After ROC analysis, these metabolites showed high sensitivity and specificity for HCC diagnosis (89). Fitian et al*.* conducted GC/LC-MS-based analysis to expand detecting scale for metabolites to characterize serum metabolic disturbances in HCV-related HCC. This work included 30 HCC patients, whose cancer arose exclusively from HCV-cirrhosis and 27 HCV patients for analysis. Among 485 detected metabolites, 107 metabolites were markedly altered in HCV-infected HCC serum compared with HCV patients. The random forest supervised class prediction model, fold difference comparisons and ROC analysis were then performed to identify metabolite expression trends that were most closely associated with HCC. Results showed that elevated serum levels of 12-hydroxyeicosatetraenoic acid, sphingosine, xanthine, serine, glycine, aspartate, and acylcarnitine were positively associated with the development of HCV-mediated HCC (90). Additionally, a metabolomics study aimed to decipher metabolic differences between HCC and HCV-related liver cirrhosis. The plasma from 44 patients were collected for GC-MS-based analysis. The MS platform detected 61 signals, 5 of which showed the strongest discriminatory capacity for separation between HCC and HCV-related cirrhosis patients. The plasma levels of oleic acid, octanoic acid, oleic acid, and glycine were highly increased in HCC, whereas the level of capric acid was decrease (91). Based on these metabolomics studies, metabolites in blood were characterized as promising biomarkers for hepatitis-related HCC.

Patients with liver cirrhosis are considered as one of the main risk factors for HCC incidence. Early diagnosis of potential HCC patients from cirrhosis patients is an urgent need in clinic. Therefore, some metabolomics studies were conducted to characterize the metabolic differences between HCC and liver cirrhosis. Using UPLC/GC-MS-based metabolomics analysis, Patterson et al*.* identified 12 up-regulated and 8 down-regulated metabolites in HCC plasma compared with liver cirrhosis patients. Among them, levels of glycodeoxycholate, deoxycholate 3-sulfate and bilirubin were significantly increased in HCC plasma compared with liver cirrhosis (92). These altered metabolites in plasma can deepen our understanding of HCC pathobiology. Besides, a GC-MS-based metabolomics study was performed in 89 plasma samples from Egyptian HCC patients and cirrhosis patients. A total of 23 differential metabolites were identified by TOF/Q-MS-based analysis in discovery cohort, which were subsequently validated by targeted metabolomics analysis. The results confirmed significant up-regulation of glutamic acid, lactic acid, valine, isoleucine, leucine, α-tocopherol, and cholesterol in HCC plasma. Notably, activation of branched-chain amnio acid metabolism was found to be associated with HCC occurrence (93). However, the limited subject was the main shortcoming of this study. A large-scale metabolomics study using 1,448 serum samples has been conducted to compare the metabolic differences between HCC and cirrhosis patients. These samples were divided into discovery (108 populations), test (684 populations), and validation (656 populations) groups. Moreover, samples from small HCC patients were specifically recruited to access the performance of potential biomarkers in early diagnosis. A total of 17 metabolites were identified with significant alterations in the HCC serum compared to liver cirrhosis group by LC-MS-based pseudo-targeted analysis. After validation, the combination of phenylalanyl-tryptophan and glycocholate, decreased in serum levels, exhibited favorable diagnostic performance for HCC, especially small HCC (94). Using the similar strategy, Kim et al*.* recruited 150 people (53 HCC patients, 47 liver cirrhosis patients, and 50 healthy volunteers) for discovery and 162 people (82 HCC patients and 80 liver cirrhosis patients) for validation. In total, 188 metabolites were detected based on GC/LC-MS-based metabolomics analysis. After evaluation in test group, increased methionine, proline, and ornithine, as well as decreased pimelylcarnitine and octanoylcarnitine in serum performed well as biomarkers to identify HCC patients from liver cirrhosis populations (95). Besides, two UPLC-MS-based metabolomics studies were conducted to identify biomarkers in serum for HCC patients. Ressom et al*.* analyzed 262 serum samples in three experiments using UPLC-QTOF-MS under positive and negative detection modes. A total of 37 dysregulated metabolites were identified. Metabolism pathway analysis showed that sphingolipid and phospholipid metabolism were enhanced, and bile acid biosynthesis was weakened in HCC cases compared to cirrhosis patients (96). Xiao et al*.* conducted UPLC-MS-based metabolomics, and identified 34 significantly altered metabolites in HCC serum in discovery group. Subsequently, a subset of these metabolites, such as Phe-Phe, glycholic acid, glycodeoxycholic acid, 3β, 6β-dihydroxy-5β-cholan-24-oic acid, and oleoyl carnitine, were verified by targeted analysis in validation group. Targeted profiling showed that bile acid-related metabolites and long chain carnitine were significantly decreased in HCC serum compared with liver cirrhosis patients. Moreover, the serum levels of glycholic acid, glycodeoxycholic acid, 3β, 6β-dihydroxy-5β-cholan-24-oic acid, and oleoyl carnitine were remarkable decreased in HCC (20). These two studies both demonstrated the potential of bile acid-related metabolites as biomarkers to distinguish HCC and cirrhosis patients. Multiple types of analytic platforms have been employed to identify metabolite biomarkers including NMR, LC-MS, CE-MS, and GC-MS. A metabolomics study employed both NMR and LC-MS devices to expand the detection scale. In this study, totally 32 differential metabolites were selected to evaluate the feasibility for HCC diagnosis by using 43 HCC serum samples and 42 liver cirrhosis serum samples. Formate and phytosphingosine were validated as a panel of biomarkers for their high sensitivity and accuracy (97). In addition, Zeng et al*.* used CE-MS-based approach to discovery the difference of serum metabolome in HCC and liver cirrhosis patients. 53 metabolites were observed with significant alterations in serum specimens. Among them, the serum levels of tryptophan and glutamine were decreased in HCC, whereas the level of 2-hydroxybutyric acid was increased. This study showed the potential of CE-MS-based approach in identifying biomarkers for HCC diagnosis (98). Apart from using biomarkers alone, the combination of metabolites and clinical covariates can act as effective approach for distinguishing HCC from liver cirrhosis. Poto et al*.* employed plasma samples from 63 HCC and 65 liver cirrhosis patients. Using GC-MS-based analysis, peak signals of 46 metabolites were obtained, 11 of which were significantly dysregulated in HCC cases. HCC cases showed higher levels of valine, serine, isoleucine, α-D-glucosamine-1-phosphate, and linoleic acid, while cirrhotic patients had elevated levels of glycine, creatinine, glutamic acid, tagatose, lauric acid, and phosphoric acid in plasma. Integrated ROC analysis showed the higher sensitivity and specificity of the combination of these metabolites and clinical covariates as an effective approach for early diagnosis than using identified metabolites or AFP alone. This work demonstrated the value of combining these metabolites with clinical covariates for early diagnosis of HCC with cirrhosis (99). Moreover, alcoholic cirrhosis is the common risk factor for HCC in western world. A ^1^H-NMR-based study profiled the metabolic features of HCC with cirrhosis compared to alcoholic cirrhosis patients by using 158 serum samples. In discovery set, the serum levels of glutamate, acetate, and N-acetylglycoproteins were remarkably increased in HCC, especially small HCC, whereas the levels of lipids and glutamine were decreased. Validation in the test set showed that the model could predict cirrhosis or HCC (100).

### Metabolite biomarkers for HCC therapeutic prediction

Metabolomics strategies have great advantages for HCC therapeutic prediction. For example, a GC-MS-based metabolomics study was performed to analyze the metabolic profiling of 130 paired HCC and normal tissues. After false discovery rates correction, 81 metabolites (21 higher and 60 lower in HCC) were identified as differential metabolites by paired nonparametric tests. Among them, FFAs, such as palmitoleic acid, palmitelaidic acid, 2-hydroxyglutaric acid, O-phosphocolamine, and elaidic acid were most increased metabolites in HCC tissues. HCC prognosis risk stratification was then performed by nonnegative matrix factorization clustering based on differential metabolites between tumor and normal tissues. This work showed that patients with higher FFAs levels had a worse prognosis (101). In addition, a LC-MS-based nontargeted and targeted metabolomics study was conducted to characterize HCC metabolic features and identify potential biomarkers for prognostic evaluation by incorporating both tissue and blood metabolites. Among 138 altered metabolites, retinol and retinal could independently differentiate HCC and cirrhosis. Using univariate and multivariate cox regression analysis, low abundance of retinol and retinal was identified as important predictors for decreased survival time (102). These studies demonstrate that metabolomics appears to have considerable value for HCC therapeutic prediction.

Liver transplantation is an effective curative therapy with long-term outcomes (103). A UPLC-MS-based metabolomics analysis aimed to explore effective biomarkers for evaluating the recurrence of HCC post-transplant. Metabolic profiling was performed on 122 HCC patients post-transplant, 25 liver cirrhosis patients, and 52 healthy individuals. Univariate analysis identified 5 differential metabolites related to HCC recurrence after liver transplantation. In detail, the increased plasma level of phosphatidylcholine (18:2/OH-16:0) as well as decreased levels of nutriacholic acid, phosphatidylcholine (o-16:0/20:4), and 2-oxo-4-methylthiobutanoic acid in HCC post-transplant were highly associated with tumor recurrence and worse outcomes (104). Apart from liver transplantation, image-guided ablation, such as transcatheter arterial chemoembolization (TACE) and radiofrequency ablation (RFA), is an alternative therapy for small and located HCC nodules (105). Two independent ^1^H-NMR metabolomics studies have been conducted in TACE-treated patients for the identification of HCC prognostic biomarkers. The first study recruited 60 HCC patients to identify serum metabolites that were associated with therapeutic outcomes of HCC after TACE treatment. It was found that higher levels of total cholesterol, low density lipoprotein cholesterol, and low-density lipoprotein particles were associated with a poorer overall survival (106). Another ^1^H-NMR-based study aimed to investigate the metabolic alterations in plasma of recurrent or metastatic HCC patients after TACE treatment or post-surgery. Increased glucose consumption and lactate and pyruvate production were observed in both TACE and post-surgery plasma compared with healthy controls. However, TACE or surgical treatments did not immediately change the metabolic features of HCC patients (19). Additionally, a ^1^H-NMR-based metabolomics also failed to generate the considerable results to distinct metabolic difference after RFA treatment, which might be due to the low sensitivity of the ^1^H-NMR platform (107). Interestingly, a GC-MS-based metabolomics study performed well in determining the candidate biomarkers for the prediction of HCC recurrence after RFA treatment. In this study, serum samples from 11 recurrent and 21 non-recurrent HCC patients after RFA were analyzed. The result showed that the combination of aspartate and glutamate can identify HCC patients for RFA treatment, whereas elevated levels of glycerol and proline can predict better patient outcomes after RFA treatment (108). Therefore, metabolomics analysis plays an important role in biomarker discovery for HCC prognostic evaluation.

### Tissue-based metabolite biomarkers for HCC development and progression

Characterizing metabolic anomalies in HCC nodules promotes the understanding of regulatory mechanisms involved in HCC development and progression. For example, a ^1^H-NMR-based metabolomics study aimed to identify metabolic preferences of HCC according of fibrosis levels. Fifty-two pairs of HCC tissues, including 26 HCC developed in severe fibrosis and 26 HCC in mild fibrosis, were sampled. A total of 34 metabolites have been observed with significant difference in different fibrosis degrees of HCC tissues. Among them, the abundance of glucose, choline derivatives, phosphoethanolamine, monounsaturated fatty acid, and triacylglyceride were detected with the most significant alterations. In detail, the results demonstrated that HCC tissues with mild fibrosis exhibited higher levels of choline derivatives and glutamine, whereas HCC tissues with severe fibrosis were characterized with decreased monounsaturated fatty acid (109). Similarly, a GC-MS-based metabolomics study provided a landscape of the metabolic differences in HCC patients with or without diabetes. Metabolic profiling was conducted for 120 diabetes or non-diabetes HCC issues. Using PCA-based analysis for selection, 2-hydroxystearate was only up-regulated in HCC tissues with diabetes, and was positively correlated with the blood glucose levels (75). These works emphasize the importance of metabolomics analysis on deciphering mechanisms of hepatocarcinogenesis in the metabolic perspective. Moreover, profiling metabolome of HCC tumors tissues *in situ* and metastases tissues can characterize the metabolism features of tumors with metastasis. Therefore, Wang et al*.* performed metabolic profiling of HCC metastasis using DEN-induced rat model with lung metastasis. After metabolic profiling, tumor tissue from metastasis sites showed decreased glucose and glycogen, and increased choline, glycine, alanine, and lactate. This ^1^H-NMR-based metabolomics analysis showed that elevated glycolysis was associated with HCC invasion and metastasis (110).

Some metabolomics studies identified metabolite biomarkers for HCC based on both serum and tissue samples. For example, Han et al*.* carried out LC-MS-based metabolomics analysis to select metabolites, the levels of which were alerted in both HCC tissue and serum samples. After integrated analysis, the abundance of chenodeoxycholic acid, lysophosphatidylcholine, and glycocholic acid were increased in HCC patients, whereas the levels of succinyladenosine and uridine were decreased. The combination of these differential metabolites can be used as a biomarker panel to improve the diagnostic accuracy of HCC (33). The identification of specific biomarkers, which are accessible in peripheral circulation can improve the diagnostic efficiency of HCC. A LC/GC-based nontargeted metabolomics analysis firstly employed 50 pairs of HCC tissues and distal non-cancerous tissues samples for global metabolic profiling. A series of 62 metabolites were observed with significant alterations in HCC tissues. Specifically, the levels of acetyl-carnitine correlated most strongly with tumor grade and could distinguish HCC and matched normal tissues. Subsequently, a targeted metabolomics was employed for external validation by using 58 serum samples (18 from HCC patients, 20 from liver cirrhosis, and 20 from healthy individuals). These findings showed that serum acetyl-carnitine was appears to be a potential biomarker for the diagnosis and progression of hepatocellular carcinoma (111). Besides, Huang et al*.* aimed to identify metabolites which were both alerted in both HCC tissues and serum samples. Using LC/GC-MS-based metabolomics analysis, 105 dysregulated metabolites in HCC tissues (30 populations) were identified compared with adjacent and distal non-tumor tissues (60 populations). These differential metabolites were then validated in HCC serum samples (30 populations) compared with serum from healthy individuals (30 populations). Through integrated analysis, the levels of betaine and propionyl-carnitine were found to be decreased in both tissues and serum (112). Previously, peripheral blood is considered unusable for investigating the mechanisms of HCC formation and development. However, a recent integrated metabolomics analysis successfully employed portal vein serum as an important sample material to identify altered metabolites as HCC progression. Portal and central vein serum, tissues, and feces samples were collected for UPLC-MS-based metabolomics analysis. Through comparing with metabolic profiling, dysregulated metabolites with the same trend in these profiling were selected for in-depth validation. In detail, higher levels of DL-3-phenyllactic acid, L-tryptophan, glycocholic acid, and 1-methylnicotinamide in portal vein serums and tissues were observed in patients with impaired liver function and poorer survival. In addition, the lower levels of linoleic acid and phenol in portal veins and feces samples were associated with higher malignant potential. This work showed that the abundance of metabolites in gut-liver axis were essential for investigating the molecular mechanism of hepatocarcinogenesis and identifying novel therapeutic targets for HCC (9). Metabolic alterations may contribute to the development of HCC, which means that the dysregulated metabolites may represent a potential target for HCC treatment.

### Multi-omics strategies containing metabolomics for biomarker discovery in HCC

Integrated omics analysis has received much attention in recent years, due to its promising values in information acquisition. Multi-omics studies can integrate multiple aspects of biological information to characterize the overall molecular information for HCC (27, 113). Recently, Le et al*.* performed a systematic multi-omics study to reveal the metabolic landscape of pro-metastatic HCC cell lines by integrating genomics, transcriptomics, proteomics, and metabolomics analysis (114). Firstly, whole-exome sequencing was performed to analysis the single-nucleotide polymorphisms and copy number alterations. Compared with low metastatic Huh7 cells, 169 and 65 nonsynonymous single-nucleotide polymorphisms, which were associated with 185 genes, were specifically observed in high metastatic MHCC97L and HCCLM3 cells. As for copy number alterations, the up-regulated alterations were observed in 19.8% and 18.8% of MHCC97L and HCCLM3 regions, while the down-regulated alterations were detected in 13.6% and 7.1% of MHCC97L and HCCLM3 regions. These altered regions were involved in 2,449 genes. Then, 9,635 differentially expressed mRNAs were detected by microarray-based transcriptomics analysis. For proteomics analysis, robust HPLC-MS/MS-based system was combined with triplex demethylation isotopic labeling approach for quantitative analysis. As a result, 737 dysregulated proteins were identified. Specifically, 311 overlapped differential genes at both mRNA and protein levels were identified in total. As for metabolomics profiling, LC-MS-based metabolomics identified 287 altered metabolites in highly metastatic HCC cells compared with HCC cells with low metastatic capacity. Glycolysis, starch and sucrose metabolism, and glutathione metabolism were up-regulated in highly metastatic HCC cells. After integrated analysis of multi-omics data and validation through western blot assay, 12 out of 311 identified genes were dysregulated at multiple biological levels at the same time. Among these selected genes, uridine diphosphate glucose pyrophosphorylase 2 (UGPase 2) in glycogen metabolism was positively correlated with the quantified metastatic capability at both mRNA and protein levels with the highest coefficient. The multi-omics data showed that UGPase 2 played an important role in promoting metastasis through the up-regulation of glycogen synthesis, and was verified as a promising pro-metastasis biomarker (114). This work demonstrates the importance of multi-omics-based analysis in biomarker discovery for HCC diagnosis and therapy. However, this study employed HCC cell lines as sample types, lacking typical pathological features of HCC tumors in clinic. Multi-omics studies are urgently required to be applied for HCC biomarker discovery using clinical materials, such as tissues or blood, in the near future.

In addition, some studies integrated data from metabolomics and transcriptomics analysis for uncovering HCC metabolic/transcriptional features. For example, in order to uncover subtypes of HCC by metabolomics-based classification, a recent multi-omics-based study firstly employed metabolomics and transcriptomics analysis on 77 tumor and paired adjacent non-cancerous tissues from HCC patients. LC/GC-MS-based metabolic profiling identified a total of 69 most significantly differential metabolites, such as nicotinamide riboside, 4-hydroxyglutamate, glutathione, sphingosine, and mono-saccharide. Then PCA-based analysis of metabolomics data showed that the normal tissues were clustered together, whereas the HCC tissues were divided into 3 subgroups (S1, S2, and S3), whose survival and clinical parameters are significantly different from each other. For in-depth analysis, subtype-specific genes were further selected for each subgroup. In detail, S1 subgroup, with a relatively poor prognosis, was characterized by a low concentration of the degradation products of phosphatidylcholine and phosphatidylethanolamine, as well as up-regulated genes on chromosome 6q27. S2 subgroup, whose prognosis was the best, showed lower levels of mono-saccharide and phosphate type, as well as few alterations in genic expressions. Finally, S3 subgroup had higher levels of unsaturated fatty acid metabolites and the worst survival outcomes (115). This metabolite-based classification provides a stable and reproducible classification method that can help predict the prognosis and prospective therapies of HCC patients. Besides, a multi-omics study employed both GC-MS-based metabolomics and transcriptomics analysis to link the metabolic perturbations with transcription classification in HCC. A panel of 30 paired tumor and non-cancerous tissues were collected for integrated analysis. The levels of glucose, glycerol-3-phosphate, glycerol-2-phosphate, malate, and linoleic acid were identified with 2-fold up-regulation in HCC tissues. This study yielded precise biochemical remodeling of HCC development, including increased glycolysis and fatty acid catabolism. As for unsupervised transcriptome analysis, transcriptomics data showed 6 subgroups of HCC (G1-G6). In detail, G1 subgroup was associated with low copy number of HBV. G2 subgroup was characterized with a high copy number of HBV. G3 subgroup was typified by mutation of *tumor protein p53* (*TP53*) and overexpression of genes controlling the cell cycle. G4 subgroup was characterized with the mutation of *transcription factor 1* (*TCF1*). G5 and G6 subgroups were strongly related to beta-catenin mutations that lead to Wnt pathway activation (116). However, HCC subgroups by transcriptomics-based classification failed to be correlated with altered metabolites (117). Another integrated study conducted LC/GC-MS-based metabolomics and RNA microarray-based transcriptomics to identify lipid biomarkers associated with HCC progression by using HCC tissues (30 populations) and healthy people (30 populations). This integrated study identified 28 metabolites and 169 genes that were significantly associated with aggressive HCC. Among them, stearoyl-CoA-desaturase (SCD) and its product, monounsaturated palmitic acid, were markedly increased in HCC tissues, and demonstrated poor prognosis of HCC patients (118). Compared with using metabolomics alone, integrated omics analysis can provide more comprehensive and accurate information associated with HCC progression.

The alterations of metabolomics profiles may be attributed to the different expression of key enzymes in metabolic pathways. Therefore, integrated analysis of proteomics and metabolomics data can enable the understanding of overall perspective of metabolic activities in HCC patients. For example, a multi-omics-based analysis aimed to investigate the biological function of HBV core protein (HBc) in HCC occurrence and development. Firstly, the differentially expressed proteins were screened by LC-MS-based proteomics. A total of 165 dysregulated proteins were detected, of which 84 proteins were up-regulated and 81 were down-regulated in HBc-overexpressing HepG2 cells. Gene Ontology (GO) enrichment analysis showed that the up-regulated proteins, with a clear trend of metabolic clustering, were mainly associated with glycolysis, glycine metabolism, and phenylalanine and tyrosine metabolism pathways, indicating the influence of HBc in HCC metabolism. Then, metabolomics data were acquired for enrichment analysis by NMR-based platform. The relatively higher levels of lactate, glutathione, phosphocholine and a range of amino acids were detected in HBc-overexpressing cells. The up-regulated metabolites were mainly clustered into glycolysis and amino acid metabolism, which was consistent with proteomics data. Finally, co-immunoprecipitation coupled with qualitative proteomics were employed to identify the interacting proteins of HBc. The results indicated that HBc regulated glycine and phenylalanine metabolism by directly binding with multiple enzymes in related pathways, including glycine N-methyltransferase, sarcosine dehydrogenase, phenylalanine hydroxylase, and 4-hydroxyphenylpyruvate dioxygenase. Besides, HBc could also interact with max-like protein X (a transcription factor) to upregulate key enzymes in glycolysis pathway. Therefore, this study exhibited a global insight into the function of HBc in HCC (119). In addition, a recent study employed proteomics and metabolomics analysis to investigate biological alterations involved in sorafenib resistance in HCC. By employing UPLC-MS-based metabolomics analysis, 26 metabolites were discovered with significantly increased abundance, such as uridine 5′-monophosphate, adenosine monophosphate, guanosine monophosphate, adenine, and cytosine. Whereas L-arginine was the only decreased metabolites in sorafenib-resistant Hep3B cells. In UPLC-MS-based proteomics analysis, 730 proteins were identified, 18 of which were significantly changed. Among them, 13 proteins were down-regulated in -resistant cells compared to parental cells, whereas 5 were up-regulated. Finally, all these dysregulated proteins/metabolites were included for a joint pathway analysis to integrate the metabolomics and proteomics profiles. The enrichment analysis revealed several dysregulated pathways involved in sorafenib-resistance, including the antifolate resistance pathway, amino acid metabolic pathway, pathways related to the protein synthesis, and other energy production metabolism. Together, this work identified potential biomarkers and therapeutic targets for sorafenib-resistant HCC (120).

In addition, a multi-omics studies firstly employed microbiomics, metabolomics, and proteomics strategies to clarify the underlying mechanisms of progression from liver cirrhosis to HCC. Firstly, 16S rRNA sequencing-based microbiomics was conducted to uncover the bacterial metataxonomic signatures for HCC and cirrhosis tissues by using 9 HCC tissues, 4 distal non-cancerous tissues, and 11 liver cirrhosis tissues. The highest LDA scores were identified for *Elizabethkingia* in HCC tissues, *Subsaxibacter* in liver cirrhosis tissues, and *Stenotrophomon* in distal non-cancerous tissues. Then, plasma (from 27 HCC patients, 30 healthy volunteers, and 23 liver cirrhosis patients) and tissues (46 from HCC patients and 30 from liver cirrhosis patients) samples were separately collected for UPLC-MS-based metabolomics analysis. Through integrating differential metabolites from plasma and tissues profiles, 16 specific metabolites were identified as dysregulated metabolites in HCC patients. 2E-eicosenoic acid and L-threonate were up-regulated in both HCC plasma and tissues, whereas betaine, choline, L-pyroglutamic acid, and phthalic acid mono-2-ethylhexyl ester were down-regulated. Besides, LC-MS-based proteomics analysis was conducted to discovery the dysregulated proteins. Compared to liver cirrhosis group, there were 107 up-regulated proteins and 137 down-regulated proteins in HCC tissues. Specifically, a combined analysis of metabolomics and proteomics by using Kyoto Encyclopedia of Genes and Genomes pathway (KEGG) analysis showed that vitamin B6 metabolic pathway was the significantly dysregulated metabolic pathway in HCC compared with liver cirrhosis. This work firstly employed microbiomics strategy for HCC multi-omics analysis, which may provide new insights into the early diagnosis, monitoring and treatment of HCC with liver cirrhosis (121).

## Conclusions and future perspective

In this review, we summarized the current workflow of metabolomics analysis and introduced the recent advances of HCC biomarkers discovery by metabolomics analysis. Accumulated evidence shows that specific metabolites in biofluids have great potential for early diagnosis, prognostic evaluation, and prediction of therapeutic outcomes. Notably, multi-omics-based strategies containing metabolomics have been successfully conducted for the discovery of metabolic biomarkers for HCC. It is through these metabolomics studies that our understanding of HCC metabolism has improved significantly.

Over the past decades, metabolomics has exhibited an increasingly important role in cancer biomarker discovery. In clinical studies, metabolomics has provided a cost-effective and productive route for biomarker discovery, which has advantages of low invasion, low sample costs, and robust reliability. Moreover, benefited from the improvement of analytical technologies, more novel metabolites can be covered and identified. However, there are still a large number of trace metabolites being ignored due to technique issues. Besides, sample type is an essential influence factor for metabolomics analysis. Some studies employed tumor cells as research objects. Nevertheless, cell lines cannot well mirror the *bona fide* metabolic features of HCC patients. Importantly, liver is a specific organ, whose blood supply is influenced by gut-liver axis interactions. Therefore, it is worth in-depth investigation of the metabolites generated from gut for HCC biomarker discovery in the future. In addition to endogenous metabolites, exogenous metabolites derived from environmental factors, such as drugs and microbe-generated metabolites, can be detected to evaluate HCC states. Last but not least, most metabolomics studies are carried out without absolute quantification of metabolites. To become reliable and robust tools for guiding clinical practice, these biomarkers need to be validated by targeted metabolomics or related biochemical analysis. Multi-omics strategies have been constantly increasing recently. Tracking biological activities by multiple types of omics technologies can provide more comprehensive and accurate information of metabolic alterations for disease progression than using metabolomics alone. However, the large volume and diversity of multi-omics data make the data integration difficult. Even worse, some emerging omics methods are lack of robust database for integrated analysis, such as metabolome. Therefore, one of the main problems of multi-omics analysis is how to truly integrate multiple omics data. KEGG or other biological pathway-based annotation can simplify analysis and map to certain pathway. Other novel and effective analytical tools for annotation or correlation analysis need to be developed for integrated analysis of multi-omics study.

Some metabolites have been identified as promising biomarkers for HCC, which are implicated in several dysregulated metabolic pathways. Metabolites in peripheral circulation are easily accessed, which can provide timely information of how metabolism is undergoing in tumor, even though for small HCC nodules. These biomarkers generated from metabolomics studies have great potential to optimize HCC therapy in clinical practice. In fact, how to translate these metabolite biomarkers in clinical practice still remains as an essential step. It has been reported that some metabolites can be accessed *in-situ* by imaging-based approaches for HCC early diagnosis or subtype classification. Therefore, in conjunction of metabolomics analysis with imaging approaches, isotope labeling methods, and derivatization-based labeling methods will accelerate the biomarker discovery and application in clinical practice. Therefore, subsequently studies need to be highlighted the validation and translation of promising biomarkers into clinical practice. In addition, most present studies aimed to identified known metabolites as biomarkers for diagnosis and prognosis, which have been discovery before. There are many unknown metabolites needed to be characterized during HCC development. Some metabolites have potential to be treated as novel therapeutic targets in clinical practice. Nowadays, metabolomics-based strategies are proposed to become an essential part of hypothesis-driven studies to discover functionally associated molecules or drugs for HCC.

Precision medicine is emerging as a tailored therapeutic strategy for individual patients based on our increased understanding of pathophysiological knowledge. Currently, metabolomics faces both opportunities and challenges in HCC precision medicine. Metabolite biomarkers, identified from HCC metabolomics studies, are regarded as one of the major components of HCC precision medicine. These potential biomarkers can be selectively employed for different clinical applications, including early diagnosis, prognostic evaluation, the prediction for therapeutic outcomes, and characterization of HCC metabolic signatures. Moreover, metabolomics analysis can uncover the metabolic status of patients, which can be utilized to guide the treatment choices and reflect the effect of current therapy. In addition, the effect of gut microflora for patients is a new dimension of HCC precision medicine. Characterizing microbe-generated metabolites by metabolomics analysis is being expected to promote the development in HCC precision medicine. However, metabolomics for precision medicine requires specialized analytical platforms for the generated big data. Comprehensive and effective medical databases incorporating metabolomics data need to be developed for integrated large-scale data processing. Besides, to increase the accessibility of metabolomics approach, metabolomics methods need to be far more standardized. Currently, precision medicine is still in its infancy. Promising metabolite biomarkers need to be applicated into clinical practice to promote the development of precision medicine. With the conducting of large scale and in-depth clinical metabolomics studies and applicating of metabolite biomarkers, metabolomics will play an increasingly important role for HCC precision medicine in the near future.

## References

[1] Bray F Ferlay J Soerjomataram I Siegel RL Torre LA Jemal A. Global cancer statistics 2018: globocan estimates of incidence and mortality worldwide for 36 cancers in 185 countries. CA Cancer J Clin. (2018) 68(6):394–424. 10.3322/caac.21492 30207593

[2] Llovet JM Kelley RK Villanueva A Singal AG Pikarsky E Roayaie S Hepatocellular carcinoma. Nat Rev Dis Primers. (2021) 7(1):6. 10.1038/s41572-020-00240-3 33479224 PMC13537433

[3] Ghouri YA Mian I Rowe JH. Review of hepatocellular carcinoma: epidemiology, etiology, and carcinogenesis. J Carcinog. (2017) 16:1. 10.4103/jcar.JCar_9_16 28694740 PMC5490340

[4] Ogunwobi OO Harricharran T Huaman J Galuza A Odumuwagun O Tan Y Mechanisms of hepatocellular carcinoma progression. World J Gastroenterol. (2019) 25(19):2279–93. 10.3748/wjg.v25.i19.2279 31148900 PMC6529884

[5] El-Serag HB. Epidemiology of viral hepatitis and hepatocellular carcinoma. Gastroenterology. (2012) 142(6):1264–73.e1. 10.1053/j.gastro.2011.12.061 22537432 PMC3338949

[6] Galun D Mijac D Filipovic A Bogdanovic A Zivanovic M Masulovic D. Precision medicine for hepatocellular carcinoma: clinical perspective. J Pers Med. (2022) 12(2):149. 10.3390/jpm12020149 35207638 PMC8879044

[7] Kim H Lee DS An TH Park HJ Kim WK Bae KH Metabolic spectrum of liver failure in type 2 diabetes and obesity: from nafld to nash to HCC. Int J Mol Sci. (2021) 22(9):4495. 10.3390/ijms22094495 PMC8123490 33925827

[8] Yang JD Hainaut P Gores GJ Amadou A Plymoth A Roberts LR. A global view of hepatocellular carcinoma: trends, risk, prevention and management. Nat Rev Gastroenterol Hepatol. (2019) 16(10):589–604. 10.1038/s41575-019-0186-y 31439937 PMC6813818

[9] Liu J Geng W Sun H Liu C Huang F Cao J Integrative metabolomic characterisation identifies altered portal vein serum metabolome contributing to human hepatocellular carcinoma. Gut. (2022) 71(6):1203–13. 10.1136/gutjnl-2021-325189. PMC9120406 34344785

[10] Fujiwara N Friedman SL Goossens N Hoshida Y. Risk factors and prevention of hepatocellular carcinoma in the era of precision medicine. J Hepatol. (2018) 68(3):526–49. 10.1016/j.jhep.2017.09.016 28989095 PMC5818315

[11] Wishart DS. Emerging applications of metabolomics in drug discovery and precision medicine. Nat Rev Drug Discov. (2016) 15(7):473–84. 10.1038/nrd.2016.32 26965202

[12] Forner A Reig M Bruix J. Hepatocellular carcinoma. Lancet. (2018) 391(10127):1301–14. 10.1016/s0140-6736(18)30010-2 29307467

[13] Marrero JA. Surveillance for hepatocellular carcinoma. Clin Liver Dis. (2020) 24(4):611–21. 10.1016/j.cld.2020.07.013 33012448

[14] Zhou J Sun H Wang Z Cong W Wang J Zeng M Guidelines for the diagnosis and treatment of hepatocellular carcinoma (2019 edition). Liver Cancer. (2020) 9(6):682–720. 10.1159/000509424 33442540 PMC7768108

[15] Girotra M Soota K Dhaliwal AS Abraham RR Garcia-Saenz-de-Sicilia M Tharian B. Utility of endoscopic ultrasound and endoscopy in diagnosis and management of hepatocellular carcinoma and its complications: what does endoscopic ultrasonography offer above and beyond conventional cross-sectional imaging? World J Gastrointest Endosc. (2018) 10(2):56–68. 10.4253/wjge.v10.i2.56 29467916 PMC5807886

[16] Marrero JA Feng Z Wang Y Nguyen MH Befeler AS Roberts LR Alpha-fetoprotein, des-gamma carboxyprothrombin, and lectin-bound alpha-fetoprotein in early hepatocellular carcinoma. Gastroenterology. (2009) 137(1):110–8. 10.1053/j.gastro.2009.04.005 19362088 PMC2704256

[17] Singal A Volk ML Waljee A Salgia R Higgins P Rogers MA Meta-analysis: surveillance with ultrasound for early-stage hepatocellular carcinoma in patients with cirrhosis. Aliment Pharmacol Ther. (2009) 30(1):37–47. 10.1111/j.1365-2036.2009.04014.x 19392863 PMC6871653

[18] Schmidt DR Patel R Kirsch DG Lewis CA Vander Heiden MG Locasale JW. Metabolomics in cancer research and emerging applications in clinical oncology. CA Cancer J Clin. (2021) 71(4):333–58. 10.3322/caac.21670 33982817 PMC8298088

[19] Chen Y Zhou J Li J Feng J Chen Z Wang X. Plasma metabolomic analysis of human hepatocellular carcinoma: diagnostic and therapeutic study. Oncotarget. (2016) 7(30):47332–42. 10.18632/oncotarget.10119 27322079 PMC5216945

[20] Xiao JF Varghese RS Zhou B Nezami Ranjbar MR Zhao Y Tsai TH Lc-Ms based serum metabolomics for identification of hepatocellular carcinoma biomarkers in egyptian cohort. J Proteome Res. (2012) 11(12):5914–23. 10.1021/pr300673x 23078175 PMC3719870

[21] Noreldeen HAA Liu X Xu G. Metabolomics of lung cancer: analytical platforms and their applications. J Sep Sci. (2020) 43(1):120–33. 10.1002/jssc.201900736 31747121

[22] Martias C Baroukh N Mavel S Blasco H Lefèvre A Roch L Optimization of sample preparation for metabolomics exploration of urine, feces, blood and saliva in humans using combined NMR and UHPLC-HRMS platforms. Molecules. (2021) 26(14):4111. 10.3390/molecules26144111 34299389 PMC8305469

[23] Jacob M Lopata AL Dasouki M Abdel Rahman AM. Metabolomics toward personalized medicine. Mass Spectrom Rev. (2019) 38(3):221–38. 10.1002/mas.21548 29073341

[24] Collins DC Sundar R Lim JSJ Yap TA. Towards precision medicine in the clinic: from biomarker discovery to novel therapeutics. Trends Pharmacol Sci. (2017) 38(1):25–40. 10.1016/j.tips.2016.10.012 27871777

[25] Kaushik AK DeBerardinis RJ. Applications of metabolomics to study cancer metabolism. Biochim Biophys Acta Rev Cancer. (2018) 1870(1):2–14. 10.1016/j.bbcan.2018.04.009 29702206 PMC6193562

[26] Olivier M Asmis R Hawkins GA Howard TD Cox LA. The need for multi-omics biomarker signatures in precision medicine. Int J Mol Sci. (2019) 20(19):4781. 10.3390/ijms20194781 PMC6801754 31561483

[27] Chen F Wang J Wu Y Gao Q Zhang S. Potential biomarkers for liver cancer diagnosis based on multi-omics strategy. Front Oncol. (2022) 12:822449. 10.3389/fonc.2022.822449 35186756 PMC8851237

[28] Kohler I Hankemeier T van der Graaf PH Knibbe CAJ van Hasselt JGC. Integrating clinical metabolomics-based biomarker discovery and clinical pharmacology to enable precision medicine. Eur J Pharm Sci. (2017) 109s:S15–S21. 10.1016/j.ejps.2017.05.018 28502671

[29] Johnson CH Ivanisevic J Siuzdak G. Metabolomics: beyond biomarkers and towards mechanisms. Nat Rev Mol Cell Biol. (2016) 17(7):451–9. 10.1038/nrm.2016.25 26979502 PMC5729912

[30] Lu W Su X Klein MS Lewis IA Fiehn O Rabinowitz JD. Metabolite measurement: pitfalls to avoid and practices to follow. Annu Rev Biochem. (2017) 86:277–304. 10.1146/annurev-biochem-061516-044952 28654323 PMC5734093

[31] El Rammouz R Létisse F Durand S Portais JC Moussa ZW Fernandez X. Analysis of skeletal muscle metabolome: evaluation of extraction methods for targeted metabolite quantification using liquid chromatography tandem mass spectrometry. Anal Biochem. (2010) 398(2):169–77. 10.1016/j.ab.2009.12.006 20026296

[32] Zhang A Sun H Wang X. Serum metabolomics as a novel diagnostic approach for disease: a systematic review. Anal Bioanal Chem. (2012) 404(4):1239–45. 10.1007/s00216-012-6117-1 22648167

[33] Han J Qin WX Li ZL Xu AJ Xing H Wu H Tissue and serum metabolite profiling reveals potential biomarkers of human hepatocellular carcinoma. Clin Chim Acta. (2019) 488:68–75. 10.1016/j.cca.2018.10.039 30389456

[34] Ivanisevic J Elias D Deguchi H Averell PM Kurczy M Johnson CH Arteriovenous blood metabolomics: a readout of intra-tissue metabostasis. Sci Rep. (2015) 5:12757. 10.1038/srep12757 26244428 PMC4525490

[35] Ganti S Weiss RH. Urine metabolomics for kidney cancer detection and biomarker discovery. Urol Oncol. (2011) 29(5):551–7. 10.1016/j.urolonc.2011.05.013 21930086 PMC3177099

[36] Zhao L Ni Y Su M Li H Dong F Chen W High throughput and quantitative measurement of microbial metabolome by gas chromatography/mass spectrometry using automated alkyl chloroformate derivatization. Anal Chem. (2017) 89(10):5565–77. 10.1021/acs.analchem.7b00660 28437060 PMC5663283

[37] Kurma K Manches O Chuffart F Sturm N Gharzeddine K Zhang J Den-induced rat model reproduces key features of human hepatocellular carcinoma. Cancers (Basel). (2021) 13(19):4981. 10.3390/cancers13194981 34638465 PMC8508319

[38] Wang KX Du GH Qin XM Gao L. 1h-Nmr-based metabolomics reveals the biomarker panel and molecular mechanism of hepatocellular carcinoma progression. Anal Bioanal Chem. (2022) 414(4):1525–37. 10.1007/s00216-021-03768-9 35024914

[39] Zeng J Huang X Zhou L Tan Y Hu C Wang X Metabolomics identifies biomarker pattern for early diagnosis of hepatocellular carcinoma: from diethylnitrosamine treated rats to patients. Sci Rep. (2015) 5:16101. 10.1038/srep16101 26526930 PMC4630653

[40] Tan Y Yin P Tang L Xing W Huang Q Cao D Metabolomics study of stepwise hepatocarcinogenesis from the model rats to patients: potential biomarkers effective for small hepatocellular carcinoma diagnosis. Mol Cell Proteomics. (2012) 11(2):M111.010694. 10.1074/mcp.M111.010694 22084000 PMC3277755

[41] Fornari F Gramantieri L Callegari E Shankaraiah RC Piscaglia F Negrini M Micrornas in animal models of hcc. Cancers (Basel). (2019) 11(12):1906. 10.3390/cancers11121906 PMC6966618 31805631

[42] He L Tian DA Li PY He XX. Mouse models of liver cancer: progress and recommendations. Oncotarget. (2015) 6(27):23306–22. 10.18632/oncotarget.4202 26259234 PMC4695120

[43] Santoni-Rugiu E Nagy P Jensen MR Factor VM Thorgeirsson SS. Evolution of neoplastic development in the liver of transgenic mice co-expressing C-myc and transforming growth factor-alpha. Am J Pathol. (1996) 149(2):407–28. 8701981 PMC1865312

[44] Jhappan C Stahle C Harkins RN Fausto N Smith GH Merlino GT. Tgf alpha overexpression in transgenic mice induces liver neoplasia and abnormal development of the mammary gland and pancreas. Cell. (1990) 61(6):1137–46. 10.1016/0092-8674(90)90076-Q 2350785

[45] Zhang X Zhu X Wang C Zhang H Cai Z. Non-targeted and targeted metabolomics approaches to diagnosing lung cancer and predicting patient prognosis. Oncotarget. (2016) 7(39):63437–48. 10.18632/oncotarget.11521 27566571 PMC5325375

[46] Dumas ME Maibaum EC Teague C Ueshima H Zhou B Lindon JC Assessment of analytical reproducibility of 1h NMR spectroscopy based metabonomics for large-scale epidemiological research: the intermap study. Anal Chem. (2006) 78(7):2199–208. 10.1021/ac0517085 16579598 PMC6561113

[47] Keun HC Ebbels TM Antti H Bollard ME Beckonert O Schlotterbeck G Analytical reproducibility in (1)H NMR-based metabonomic urinalysis. Chem Res Toxicol. (2002) 15(11):1380–6. 10.1021/tx0255774 12437328

[48] Kang C. Applications of in-cell NMR in structural biology and drug discovery. Int J Mol Sci. (2019) 20(1):139. 10.3390/ijms20010139 PMC6337603 30609728

[49] Baumstark D Kremer W Boettcher A Schreier C Sander P Schmitz G (1)H NMR spectroscopy quantifies visibility of lipoproteins, subclasses, and lipids at varied temperatures and pressures. J Lipid Res. (2019) 60(9):1516–34. 10.1194/jlr.M092643 31239285 PMC6718440

[50] Tzika AA Cheng LL Goumnerova L Madsen JR Zurakowski D Astrakas LG Biochemical characterization of pediatric brain tumors by using in vivo and ex vivo magnetic resonance spectroscopy. J Neurosurg. (2002) 96(6):1023–31. 10.3171/jns.2002.96.6.1023 12066902

[51] Choi C Raisanen JM Ganji SK Zhang S McNeil SS An Z Prospective longitudinal analysis of 2-hydroxyglutarate magnetic resonance spectroscopy identifies broad clinical utility for the management of patients with idh-mutant glioma. J Clin Oncol. (2016) 34(33):4030–9. 10.1200/JCO.2016.67.1222 28248126 PMC5477829

[52] Markley JL Brüschweiler R Edison AS Eghbalnia HR Powers R Raftery D The future of NMR-based metabolomics. Curr Opin Biotechnol. (2017) 43:34–40. 10.1016/j.copbio.2016.08.001 27580257 PMC5305426

[53] Granlund KL Tee SS Vargas HA Lyashchenko SK Reznik E Fine S Hyperpolarized MRI of human prostate cancer reveals increased lactate with tumor grade driven by monocarboxylate transporter 1. Cell Metab. (2020) 31(1):105–14.e3. 10.1016/j.cmet.2019.08.024 31564440 PMC6949382

[54] Faubert B Li KY Cai L Hensley CT Kim J Zacharias LG Lactate metabolism in human lung tumors. Cell. (2017) 171(2):358–71.e9. 10.1016/j.cell.2017.09.019 28985563 PMC5684706

[55] Tang DQ Zou L Yin XX Ong CN. HILIC-MS for metabolomics: an attractive and complementary approach to RPLC-MS. Mass Spectrom Rev. (2016) 35(5):574–600. 10.1002/mas.21445 25284160

[56] Wu H Xue R Dong L Liu T Deng C Zeng H Metabolomic profiling of human urine in hepatocellular carcinoma patients using gas chromatography/mass spectrometry. Anal Chim Acta. (2009) 648(1):98–104. 10.1016/j.aca.2009.06.033 19616694

[57] Chiu HH Kuo CH. Gas chromatography-mass spectrometry-based analytical strategies for fatty acid analysis in biological samples. J Food Drug Anal. (2020) 28(1):60–73. 10.1016/j.jfda.2019.10.003 31883609 PMC13521055

[58] Roessner U Wagner C Kopka J Trethewey RN Willmitzer L. Technical advance: simultaneous analysis of metabolites in potato tuber by gas chromatography-mass spectrometry. Plant J. (2000) 23(1):131–42. 10.1046/j.1365-313x.2000.00774.x 10929108

[59] Mirnaghi FS Caudy AA. Challenges of analyzing different classes of metabolites by a single analytical method. Bioanalysis. (2014) 6(24):3393–416. 10.4155/bio.14.236 25534794

[60] Njoku K Sutton CJ Whetton AD Crosbie EJ. Metabolomic biomarkers for detection, prognosis and identifying recurrence in endometrial cancer. Metabolites. (2020) 10(8):134. 10.3390/metabo10080314 32751940 PMC7463916

[61] Zhang J Yang S Wang J Xu Y Zhao H Lei J Integrated LC-MS metabolomics with dual derivatization for quantification of ffas in fecal samples of hepatocellular carcinoma patients. J Lipid Res. (2021) 62:100143. 10.1016/j.jlr.2021.100143 34710433 PMC8599149

[62] Büscher JM Czernik D Ewald JC Sauer U Zamboni N. Cross-Platform comparison of methods for quantitative metabolomics of primary metabolism. Anal Chem. (2009) 81(6):2135–43. 10.1021/ac8022857 19236023

[63] Wang Q Su B Dong L Jiang T Tan Y Lu X Liquid chromatography-mass spectrometry-based nontargeted metabolomics predicts prognosis of hepatocellular carcinoma after curative resection. J Proteome Res. (2020) 19(8):3533–41. 10.1021/acs.jproteome.0c00344 32618195

[64] Fenn JB Mann M Meng CK Wong SF Whitehouse CM. Electrospray ionization for mass spectrometry of large biomolecules. Science. (1989) 246(4926):64–71. 10.1126/science.2675315 2675315

[65] Koek MM van der Kloet FM Kleemann R Kooistra T Verheij ER Hankemeier T. Semi-automated non-target processing in GC×GC-ms metabolomics analysis: applicability for biomedical studies. Metabolomics. (2011) 7(1):1–14. 10.1007/s11306-010-0219-6 21461033 PMC3040320

[66] Bingol K. Recent advances in targeted and untargeted metabolomics by NMR and MS/NMR methods. High Throughput. (2018) 7(2):9. 10.3390/ht7020009 29670016 PMC6023270

[67] Oliveira RV Henion J Wickremsinhe E. Fully-automated approach for online dried blood spot extraction and bioanalysis by two-dimensional-liquid chromatography coupled with high-resolution quadrupole time-of-flight mass spectrometry. Anal Chem. (2014) 86(2):1246–53. 10.1021/ac403672u 24364804

[68] Kumar P Rúbies A Centrich F Granados M Cortés-Francisco N Caixach J Targeted analysis with benchtop quadrupole-orbitrap hybrid mass spectrometer: application to determination of synthetic hormones in animal urine. Anal Chim Acta. (2013) 780:65–73. 10.1016/j.aca.2013.04.017 23680552

[69] Hopfgartner G Varesio E Tschäppät V Grivet C Bourgogne E Leuthold LA. Triple quadrupole linear ion trap mass spectrometer for the analysis of small molecules and macromolecules. J Mass Spectrom. (2004) 39(8):845–55. 10.1002/jms.659 15329837

[70] Campbell JL Le Blanc JC. Using high-resolution quadrupole tof technology in dmpk analyses. Bioanalysis. (2012) 4(5):487–500. 10.4155/bio.12.14 22409548

[71] Khamis MM Adamko DJ El-Aneed A. Mass spectrometric based approaches in urine metabolomics and biomarker discovery. Mass Spectrom Rev. (2017) 36(2):115–34. 10.1002/mas.21455 25881008

[72] Roberts JM Dai DLY Hollander Z Ng RT Tebbutt SJ Wilcox PG Multiple reaction monitoring mass spectrometry to identify novel plasma protein biomarkers of treatment response in cystic fibrosis pulmonary exacerbations. J Cyst Fibros. (2018) 17(3):333–40. 10.1016/j.jcf.2017.10.013 29174082

[73] Shao Y Zhu B Zheng R Zhao X Yin P Lu X Development of urinary pseudotargeted LC-MS-based metabolomics method and its application in hepatocellular carcinoma biomarker discovery. J Proteome Res. (2015) 14(2):906–16. 10.1021/pr500973d 25483141

[74] Courant F Antignac JP Monteau F Le Bizec B. Metabolomics as a potential new approach for investigating human reproductive disorders. J Proteome Res. (2013) 12(6):2914–20. 10.1021/pr400204q 23651426

[75] Xia H Chen J Sekar K Shi M Xie T Hui KM. Clinical and metabolomics analysis of hepatocellular carcinoma patients with diabetes mellitus. Metabolomics. (2019) 15(12):156. 10.1007/s11306-019-1619-x 31773292

[76] Lutz U Lutz RW Lutz WK. Metabolic profiling of glucuronides in human urine by LC-MS/MS and partial least-squares discriminant analysis for classification and prediction of gender. Anal Chem. (2006) 78(13):4564–71. 10.1021/ac0522299 16808466

[77] Zhou P Zhou N Shao L Li J Liu S Meng X Diagnosis of clostridium difficile infection using an UPLC-MS based metabolomics method. Metabolomics. (2018) 14(8):102. 10.1007/s11306-018-1397-x 30830376

[78] Zhou PC Sun LQ Shao L Yi LZ Li N Fan XG. Establishment of a pattern recognition metabolomics model for the diagnosis of hepatocellular carcinoma. World J Gastroenterol. (2020) 26(31):4607–23. 10.3748/wjg.v26.i31.4607 32884220 PMC7445864

[79] Tzartzeva K Obi J Rich NE Parikh ND Marrero JA Yopp A Surveillance imaging and alpha fetoprotein for early detection of hepatocellular carcinoma in patients with cirrhosis: a meta-analysis. Gastroenterology. (2018) 154(6):1706–18.e1. 10.1053/j.gastro.2018.01.064 29425931 PMC5927818

[80] Peng L Cantor DI Huang C Wang K Baker MS Nice EC. Tissue and plasma proteomics for early stage cancer detection. Molecular Omics. (2018) 14(6):405–23. 10.1039/C8MO00126J 30251724

[81] Jee SH Kim M Kim M Yoo HJ Kim H Jung KJ Metabolomics profiles of hepatocellular carcinoma in a Korean prospective cohort: the Korean cancer prevention study-II. Cancer Prev Res (Phila). (2018) 11(5):303–12. 10.1158/1940-6207.CAPR-17-0249 29500188

[82] Hang D Yang X Lu J Shen C Dai J Lu X Untargeted plasma metabolomics for risk prediction of hepatocellular carcinoma: a prospective study in two Chinese cohorts. Int J Cancer. (2022) 151(12):2144–54. 10.1002/ijc.34229 35904854

[83] Casadei-Gardini A Del Coco L Marisi G Conti F Rovesti G Ulivi P (1)H-NMR based serum metabolomics highlights different specific biomarkers between early and advanced hepatocellular carcinoma stages. Cancers (Basel). (2020) 12(1):241. 10.3390/cancers12010241 PMC7016798 31963766

[84] Fages A Duarte-Salles T Stepien M Ferrari P Fedirko V Pontoizeau C Metabolomic profiles of hepatocellular carcinoma in a European prospective cohort. BMC Med. (2015) 13:242. 10.1186/s12916-015-0462-9 26399231 PMC4581424

[85] Zhu Q Yuan B Qiao GL Yan JJ Li Y Duan R Prognostic factors for survival after hepatic resection of early hepatocellular carcinoma in HBV-related cirrhotic patients. Clin Res Hepatol Gastroenterol. (2016) 40(4):418–27. 10.1016/j.clinre.2015.12.007 26823044

[86] Chiu CC Wang JJ Chen YS Chen JJ Tsai TC Lai CC Trends and predictors of outcomes after surgery for hepatocellular carcinoma: a nationwide population-based study in Taiwan. Eur J Surg Oncol. (2015) 41(9):1170–8. 10.1016/j.ejso.2015.04.023 26048295

[87] Gao R Cheng J Fan C Shi X Cao Y Sun B Serum metabolomics to identify the liver disease-specific biomarkers for the progression of hepatitis to hepatocellular carcinoma. Sci Rep. (2015) 5:18175. 10.1038/srep18175 26658617 PMC4674760

[88] Pan HY Wu QQ Yin QQ Dai YN Huang YC Zheng W LC/MS-based global metabolomic identification of serum biomarkers differentiating hepatocellular carcinoma from chronic hepatitis B and liver cirrhosis. ACS omega. (2021) 6(2):1160–70. 10.1021/acsomega.0c04259 33490775 PMC7818305

[89] Wei S Suryani Y Gowda GA Skill N Maluccio M Raftery D. Differentiating hepatocellular carcinoma from hepatitis C using metabolite profiling. Metabolites. (2012) 2(4):701–16. 10.3390/metabo2040701 24957758 PMC3901236

[90] Fitian AI Nelson DR Liu C Xu Y Ararat M Cabrera R. Integrated metabolomic profiling of hepatocellular carcinoma in hepatitis C cirrhosis through GC/MS and UPLC/MS-MS. Liver Int. (2014) 34(9):1428–44. 10.1111/liv.12541 24661807 PMC4169337

[91] Nomair AM Madkour MA Shamseya MM Elsheredy HG Shokr A. Profiling of plasma metabolomics in patients with hepatitis C-related liver cirrhosis and hepatocellular carcinoma. Clin Exp Hepatol. (2019) 5(4):317–26. 10.5114/ceh.2019.89478 31893244 PMC6935851

[92] Patterson AD Maurhofer O Beyoglu D Lanz C Krausz KW Pabst T Aberrant lipid metabolism in hepatocellular carcinoma revealed by plasma metabolomics and lipid profiling. Cancer Res. (2011) 71(21):6590–600. 10.1158/0008-5472.CAN-11-0885 21900402 PMC3206149

[93] Nezami Ranjbar MR Luo Y Di Poto C Varghese RS Ferrarini A Zhang C GC-MS based plasma metabolomics for identification of candidate biomarkers for hepatocellular carcinoma in egyptian cohort. PLoS One. (2015) 10(6):e0127299. 10.1371/journal.pone.0127299 26030804 PMC4452085

[94] Luo P Yin P Hua R Tan Y Li Z Qiu G A large-scale, multicenter serum metabolite biomarker identification study for the early detection of hepatocellular carcinoma. Hepatology (Baltimore, Md). (2018) 67(2):662–75. 10.1002/hep.29561 28960374 PMC6680350

[95] Kim DJ Cho EJ Yu KS Jang IJ Yoon JH Park T Comprehensive metabolomic search for biomarkers to differentiate early stage hepatocellular carcinoma from cirrhosis. Cancers (Basel). (2019) 11(10):1497. 10.3390/cancers11101497 PMC6826937 31590436

[96] Ressom HW Xiao JF Tuli L Varghese RS Zhou B Tsai TH Utilization of metabolomics to identify serum biomarkers for hepatocellular carcinoma in patients with liver cirrhosis. Anal Chim Acta. (2012) 743:90–100. 10.1016/j.aca.2012.07.013 22882828 PMC3419576

[97] Liu Y Hong Z Tan G Dong X Yang G Zhao L NMR and LC/MS-based global metabolomics to identify serum biomarkers differentiating hepatocellular carcinoma from liver cirrhosis. Int J Cancer. (2014) 135(3):658–68. 10.1002/ijc.28706 24382646

[98] Zeng J Yin P Tan Y Dong L Hu C Huang Q Metabolomics study of hepatocellular carcinoma: discovery and validation of serum potential biomarkers by using capillary electrophoresis-mass spectrometry. J Proteome Res. (2014) 13(7):3420–31. 10.1021/pr500390y 24853826

[99] Di Poto C Ferrarini A Zhao Y Varghese RS Tu C Zuo Y Metabolomic characterization of hepatocellular carcinoma in patients with liver cirrhosis for biomarker discovery. Cancer Epidemiol Biomarkers Prev. (2017) 26(5):675–83. 10.1158/1055-9965.EPI-16-0366 27913395 PMC5413442

[100] Nahon P Amathieu R Triba MN Bouchemal N Nault JC Ziol M Identification of serum proton NMR metabolomic fingerprints associated with hepatocellular carcinoma in patients with alcoholic cirrhosis. Clin Cancer Res. (2012) 18(24):6714–22. 10.1158/1078-0432.CCR-12-1099 23136190

[101] Fang C Wang H Lin Z Liu X Dong L Jiang T Metabolic reprogramming and risk stratification of hepatocellular carcinoma studied by using gas chromatography-mass spectrometry-based metabolomics. Cancers (Basel). (2022) 14(1):231. 10.3390/cancers14010231 PMC8750553 35008393

[102] Han J Han ML Xing H Li ZL Yuan DY Wu H Tissue and serum metabolomic phenotyping for diagnosis and prognosis of hepatocellular carcinoma. Int J Cancer. (2020) 146(6):1741–53. 10.1002/ijc.32599 31361910

[103] Sapisochin G Bruix J. Liver transplantation for hepatocellular carcinoma: outcomes and novel surgical approaches. Nat Rev Gastroenterol Hepatol. (2017) 14(4):203–17. 10.1038/nrgastro.2016.193 28053342

[104] Lu D Yang F Lin Z Zhuo J Liu P Cen B A prognostic fingerprint in liver transplantation for hepatocellular carcinoma based on plasma metabolomics profiling. Eur J Surg Oncol. (2019) 45(12):2347–52. 10.1016/j.ejso.2019.07.004 31331801

[105] Heimbach JK Kulik LM Finn RS Sirlin CB Abecassis MM Roberts LR Aasld guidelines for the treatment of hepatocellular carcinoma. Hepatology (Baltimore, Md). (2018) 67(1):358–80. 10.1002/hep.29086 28130846

[106] Geyer T Rübenthaler J Alunni-Fabbroni M Schinner R Weber S Mayerle J NMR-based lipid metabolite profiles to predict outcomes in patients undergoing interventional therapy for a hepatocellular carcinoma (HCC): a substudy of the SORAMIC trial. Cancers (Basel). (2021) 13(11):2787. 10.3390/cancers13112787 PMC8199928 34205110

[107] Goossens C Nahon P Le Moyec L Triba MN Bouchemal N Amathieu R Sequential serum metabolomic profiling after radiofrequency ablation of hepatocellular carcinoma reveals different response patterns according to etiology. J Proteome Res. (2016) 15(5):1446–54. 10.1021/acs.jproteome.5b01032 27015127

[108] Liu Z Nahon P Li Z Yin P Li Y Amathieu R Determination of candidate metabolite biomarkers associated with recurrence of HCV-related hepatocellular carcinoma. Oncotarget. (2018) 9(5):6245–58. 10.18632/oncotarget.23500 29464069 PMC5814209

[109] Buchard B Teilhet C Abeywickrama Samarakoon N Massoulier S Joubert-Zakeyh J Blouin C Two metabolomics phenotypes of human hepatocellular carcinoma in non-alcoholic fatty liver disease according to fibrosis severity. Metabolites. (2021) 11(1):54. 10.3390/metabo11010054 33466889 PMC7830343

[110] Wang J Zhang S Li Z Yang J Huang C Liang R (1)H-NMR-based metabolomics of tumor tissue for the metabolic characterization of rat hepatocellular carcinoma formation and metastasis. Tumour Biol. (2011) 32(1):223–31. 10.1007/s13277-010-0116-7 20890798

[111] Lu Y Li N Gao L Xu YJ Huang C Yu K Acetylcarnitine is a candidate diagnostic and prognostic biomarker of hepatocellular carcinoma. Cancer Res. (2016) 76(10):2912–20. 10.1158/0008-5472.CAN-15-3199 26976432

[112] Huang Q Tan Y Yin P Ye G Gao P Lu X Metabolic characterization of hepatocellular carcinoma using nontargeted tissue metabolomics. Cancer Res. (2013) 73(16):4992–5002. 10.1158/0008-5472.CAN-13-0308 23824744

[113] Nie W Yan L Lee YH Guha C Kurland IJ Lu H. Advanced mass spectrometry-based multi-omics technologies for exploring the pathogenesis of hepatocellular carcinoma. Mass Spectrom Rev. (2016) 35(3):331–49. 10.1002/mas.21439 24890331

[114] Li Y Zhuang H Zhang X Li Y Liu Y Yi X Multiomics integration reveals the landscape of prometastasis metabolism in hepatocellular carcinoma. Mol Cell Proteomics. (2018) 17(4):607–18. 10.1074/mcp.RA118.000586 29371291 PMC5880115

[115] Hou G Ding D Tian T Dong W Sun D Liu G Metabolomics-based classification reveals subtypes of hepatocellular carcinoma. Mol Carcinog. (2022) 61(11):989–1001. 10.1002/mc.23455 36121331

[116] Boyault S Rickman DS de Reyniès A Balabaud C Rebouissou S Jeannot E Transcriptome classification of HCC is related to gene alterations and to new therapeutic targets. Hepatology (Baltimore, Md). (2007) 45(1):42–52. 10.1002/hep.21467 17187432

[117] Beyoğlu D Imbeaud S Maurhofer O Bioulac-Sage P Zucman-Rossi J Dufour JF Tissue metabolomics of hepatocellular carcinoma: tumor energy metabolism and the role of transcriptomic classification. Hepatology (Baltimore, Md). (2013) 58(1):229–38. 10.1002/hep.26350 PMC3695036 23463346

[118] Budhu A Roessler S Zhao X Yu Z Forgues M Ji J Integrated metabolite and gene expression profiles identify lipid biomarkers associated with progression of hepatocellular carcinoma and patient outcomes. Gastroenterology. (2013) 144(5):1066–75.e1. 10.1053/j.gastro.2013.01.054 23376425 PMC3633738

[119] Xie Q Fan F Wei W Liu Y Xu Z Zhai L Multi-omics analyses reveal metabolic alterations regulated by hepatitis B virus core protein in hepatocellular carcinoma cells. Sci Rep. (2017) 7:41089. 10.1038/srep41089 28112229 PMC5253728

[120] Abushawish KYI Soliman SSM Giddey AD Al-Hroub HM Mousa M Alzoubi KH Multi-omics analysis revealed a significant alteration of critical metabolic pathways due to sorafenib-resistance in Hep3b cell lines. Int J Mol Sci. (2022) 23(19):11975. 10.3390/ijms231911975 36233276 PMC9569810

[121] Mei M Liu D Tang X You Y Peng B He X Vitamin B6 metabolic pathway is involved in the pathogenesis of liver diseases via multi-omics analysis. J Hepatocell Carcinoma. (2022) 9:729–50. 10.2147/JHC.S370255 35979344 PMC9377404

