# Supplementary material for: Metabolomics in hepatocellular carcinoma: From biomarker discovery to precision medicine
Source: Front Med Technol. 2023 Jan 4;4:1065506. doi: 10.3389/fmedt.2022.1065506 (PMC9845953; doi:10.3389/fmedt.2022.1065506)
Supplement: Supplementary file 1 [file Table1.docx]

Supplementary Table 1. Summary of recent metabolomics studies for HCC.

|  | Platforms | Specimens | Experimental group | Control group | Up-regulated metabolites | Down-regulated metabolites | References |
| --- | --- | --- | --- | --- | --- | --- | --- |
| Metabolomics studies for HCC early diagnosis | CE-MS | Serum | DEN-induced HCC (n=7) | Healthy control (n=7) | Creatine | Betaine | (39) |
|  | ^1^H-NMR | Urine | DEN-induced HCC (n=18) | Healthy control (n=18) | Creatinine, putrescine, choline, taurine | Hippurate | (38) |
|  |  | Serum | HCC serum (n=262) | Liver cirrhosis (n=76), hepatitis B (n=74) |  |  |  |
|  | LC-MS | Serum | DEN-induced HCC (n=28) | Healthy control (n=28) | Taurocholic acid, lysophosphoethanolamine, lysophosphatidylcholine | - | (40) |
|  | UPLC-MS | Serum | HCC serum (n=75) | Healthy control (n=134) | Leucine, phenylalanine, tyrosine, arachidonic acid, 5-hydroxyhexanoic acid, tauroursodeoxycholic acid | Oleamide, androsterone sulfate, lysophosphatidic acid | (81) |
|  | GC/MS | Urine | HCC urine (n=20) | Healthy control (n=20) | Octanedioic acid, glycine, L-tyrosine, L-threonine, butanedioic acid | Hypoxanthine, primidine | (56) |
|  | UPLC-MS | Plasma | HCC plasma (n=163) | Healthy control (n=163) | 16α-hydroxydehydroepiandrosterone 3-sulfate, glycocholic acid, glycochenodeoxycholic acid 3-sulfate, glycochenodeoxycholic acid 3-glucuronide, hydroxyphenyllactic acid, cystathionine, citrulline, arginine, sarcosine, quinolinate | 4-androsten-3β,17β-diol 3-sulfate, 4-androsten-3β,17β-diol sulfate, ceramide, citraconate | (82) |
|  | ^1^H-NMR | Serum | Advanced HCC (n=36) | Early HCC (n=28) | Glycine, glucose, galactose, N-acetylglycoproteins | Alanine, glutamine, 1-methylhistidine, lactate, lysine, valine | (83) |
|  | ^1^H-NMR | Serum | HCC serum (n=144) | Healthy control (n=222) | Tyrosine, phenylalanine, citrate, glucose, propylene glycol | Glutamate, leucine, isoleucine, choline, N-acetylglycoproteins, unsaturated lipids | (84) |
|  | GC-MS | Serum | HCC serum (n=39) | Healthy control (n=61), hepatitis B (n=49), cirrhosis (n=52) | Glutamate | Palmitic acid, asparagine | (87) |
|  | LC-MS | Serum | HCC serum (n=30) | Hepatitis B (n=30), liver cirrhosis (n=29) | Taurodeoxy cholic acid, 1,2-diacyl-3-β-D-galactosyl-sn-glycerol | Glycyrrhizic acid | (88) |
|  | ^1^H-NMR | Serum | HCC serum (n=40) | Hepatitis C (n=22) | Choline, valine | Creatinine | (89) |
|  | UPLC-MS, GC-MS | Serum | HCC serum (n=30) | Healthy control (n=30), HCV related cirrhosis (n=27) | 12-hydroxyeicosatetraenoic acid, sphingosine, xanthine, serine, glycine, aspartate | - | (90) |
|  | GC/MS | Plasma | HCC plasma (n=22) | HCV-related cirrhosis (n=22) | Oleic acid, octanoic acid, glycine | Capric acid | (91) |
|  | UPLC-MS, GC-MS | Plasma | HCC plasma (n=20) | Liver cirrhosis (n=7), acute myelogenous leukemia (n=22) | Glycodeoxycholate, deoxycholate 3-sulfate, bilirubin | Lignoceric acid, nervonic acid | (92) |
|  | GC-MS | Plasma | HCC plasma (n=40) | Liver cirrhosis (n=49) | Glutamic acid, lactic acid, valine, isoleucine, leucine, α-tocopherol, cholesterol | Citric acid, sorbose | (93) |
|  | LC-MS | Serum | HCC serum (n=36) | Healthy control (n=31), liver cirrhosis (n=41) | - | Phenylalanyl-tryptophan, glycocholate | (94) |
|  | LC-MS, GC-MS | Serum | Early HCC serum (n=50) | Healthy control (n=50), liver cirrhosis (n=47) | Methionine, proline, ornithine | Pimelylcarnitine, octanoylcarnitine | (95) |
|  | UPLC-MS | Serum | HCC serum (n=78) | Liver cirrhosis (n=184) | Sphingosine-1-phosphate, lysophosphatidylcholine | Glycochenodeoxycholic acid 3-sulfate, glycocholic acid, glycodeoxycholic acid, taurocholic acid, taurochenodeoxycholate | (96) |
|  | UPLC-MS | Serum | HCC serum (n=40) | Liver cirrhosis (n=49) | Phe-Phe | Glycholic acid, glycodeoxycholic acid, 3β, 6β-dihydroxy-5β-cholan-24-oic acid, oleoyl carnitine | (20) |
|  | ^1^H-NMR, LC-MS | Serum | HCC serum (n=43) | Liver cirrhosis (n=42) | Ascorbate, oxaloacetate, glycerol | Formate, tyrosine, phytosphingosine | (97) |
|  | CE-MS | Serum | HCC serum (n=22) | Healthy control (n=30), liver cirrhosis (n=25) | 2-hydroxybutyric acid | Tryptophan, glutamine | (98) |
|  | GC-MS | Plasma | HCC plasma (n=63) | Liver cirrhosis (n=65) | Glycine, pyroglutamic acid, linoleic acid | Valine, serine, isoleucine, creatinine, lauric acid, phosphoric acid | (99) |
|  | ^1^H-NMR | Serum | HCC serum (n=61) | Liver cirrhosis (n=97) | Glutamate, acetate, and N-acetylglycoproteins | Glutamine | (100) |
| Metabolomics studies for HCC therapeutic prediction | GC-MS | Tissue | HCC tissues (n=130) | Adjacent non-cancerous tissues (n=130) | Palmitoleic acid, palmitelaidic acid, elaidic acid | Oleic acid, palmitic acid, myristic acid | (101) |
|  | UPLC-MS | Tissue | HCC tissues (n=156) | Distal non-cancerous tissues (n=156) | - | Retinol, retinal | (102) |
|  | UPLC-MS | Plasma | HCC after liver transplantation (n=122) | Healthy control (n=52), liver cirrhosis (n=25) | Phosphatidylcholine | Nutriacholic acid, 2-oxo-4-methylthiobutanoic acid | (104) |
|  | ^1^H-NMR | Serum | HCC after interventional therapy (n=30) | HCC serum (n=30) | Cholesterol | - | (106) |
|  | ^1^H-NMR | Plasma | HCC plasma (n=108) | Healthy control (n=60) | Lactate, phenylalanine, hypoxanthine, pyruvate | Glucose | (19) |
|  | ^1^H-NMR | Serum | Post-radiofrequency ablation (HCC, n=120) | Pre-radiofrequency ablation (HCC, n=120) | - | - | (107) |
|  | GC-MS | Serum | Recurrent HCC patients after radiofrequency ablation (n=11) | Non-recurrent HCC patients (n=10) | Aspartate, glutamate, glycerol, proline | - | (108) |
| Metabolomics studies for HCC development and progression | ^1^H-NMR | Tissues | HCC with severe fibrosis (n=26) | HCC with mild fibrosis (n=26) | Glucose, phosphoethanolamine, triacylglyceride | Monounsaturated fatty acid | (109) |
|  | UPLC-M0S | Tissues | HCC with diabetes (n=34) | HCC without diabetes (n=26) | 2-hydroxystearate | - | (75) |
|  | ^1^H-NMR | Tissues | DEN-induced HCC with lung metastasis (n=15) | Healthy control (n=15) | Lactate, choline, glycine | Glucose | (110) |
|  | UPLC-MS | Tissues | HCC tissues (n=30) | Distal non-cancerous  tissues (n=30) | Succinyladenosine, uridine | Chenodeoxycholic acid, glycocholic acid | (33) |
|  |  | Serum | HCC serum (n=30) | Healthy control (n=30), liver cirrhosis (n=30) |  |  |  |
|  | LC/GC-MS | Tissues | HCC tissues (n=50) | Distal non-cancerous tissues (n=50) | - | Acetyl-carnitine | (111) |
|  |  | Serum | HCC serum (n=18) | Healthy control (n=20), liver cirrhosis (n=20) |  |  |  |
|  | UPLC-MS | Tissues | HCC tissues (n=50) | Adjacent non-cancerous tissues (n=50), distal non-cancerous  tissues (n=50) | - | Betaine, propionylcarnitine | (112) |
|  | UPLC-MS, GC-MS | Serum | HCC serum (n=139) | Chronic hepatitis (n=81), liver cirrhosis (n=78) |  |  |  |
|  | UPLC-MS | Central vein serum, portal vein serum, tissues, feces | HCC (n=50) | Healthy control (n=50), adjacent non-cancerous tissues (n=52) | DL-3-phenyllactic acid, glycocholic acid, 1-methylnicotinamide, L-tryptophan | Linoleic acid, phenol | (9) |
| Multi-omics-based studies containing metabolomics for HCC | Illumina-based genomics, microarray-based transcriptomics, HPLC-MS/MS-based-proteomics, LC-MS-based metabolomics | HCC Cell lines | HCCLM3 andMHCC97L cells | Huh7 cells | Maltose, glutathione disulfide, putrescine | Dihydroxyacetone phosphate, trehalose, sucrose | (114) |
|  | RNA sequencing-based transcriptomics, GC/LC‐MS-based metabolomics | Tissues | HCC tissues (n=77) | Paired adjacent non-cancerous tissues (n=77) | Nicotinamide riboside, 4‐hydroxyglutamate, deoxy-carnitine, S‐adenosylmethionine, inosine 5′‐monophosphate, guanosine 5′‐onophosphate, homoserine, asparagylleucine, adenosine 5′‐monophosphate | Glutathione, sphingosine, mono‐saccharide | (115) |
|  | LC-MS-based proteomics, ^1^H-NMR-based metabolomics | HCC Cell lines | HBV core protein overexpressing HepG2 cells | Parental HepG2 cells | Lactate, glutathione, phosphocholine, glycerophosphocholine, glycine, tyrosine, phenylalanine | - | (119) |
|  | UPLC-MS-based proteomics and metabolomics | HCC Cell lines | Sorafenib-Resistant Hep3B Cells | Parental Hep3B Cells | Uridine 5’-monophosphate, adenosine monophosphate, guanosine monophosphate, adenine, cytosine | L-arginine | (120) |
|  | 16S rRNA sequencing-based microbiomics, LC-MS-based proteomics, UPLC-MS-based metabolomics | Tissues | HCC tissues (n=46) | Liver cirrhosis (n=30) | 2E-eicosenoic acid, L-threonate | Betaine, choline, L-pyroglutamic acid, phthalic acid mono-2-ethylhexyl ester | (121) |
|  |  | Plasma | HCC plasma (n=27) | Healthy control (n=30), liver cirrhosis (n=23) |  |  |  |
|  | GC-MS, microarray-based transcriptomics | Tissues | HCC tissues (n=30) | Non-cancerous tissues (n=30) | Glucose, glycerol 3-phosphate, glycerol 2-phosphate, malate, linoleic acid | Myo-inositol, alanine | (117) |
|  | UPLC-MS, GC-MS, microarray-based transcriptomics | Tissues | HCC tissues (n=30) | Paired non-cancerous  tissues (n=30) | Monounsaturated palmitic acid | - | (118) |
